# Forecasting Human African Trypanosomiasis Prevalences from Population Screening Data Using Continuous Time Models

**DOI:** 10.1371/journal.pcbi.1005103

**Published:** 2016-09-22

**Authors:** Harwin de Vries, Albert P. M. Wagelmans, Epco Hasker, Crispin Lumbala, Pascal Lutumba, Sake J. de Vlas, Joris van de Klundert

**Affiliations:** 1 Econometric Institute, Erasmus University Rotterdam, Rotterdam, The Netherlands; 2 Department of Public Health, Institute Of Tropical Medicine, Antwerp, Belgium; 3 Programme National de Lutte contre la Trypanosomiase Humaine Africain (PNLTHA), Kinshasa, Democratic Republic of Congo; 4 University of Kinshasa, Kinshasa, Democratic Republic of Congo; 5 Department of Public Health, Erasmus MC, University Medical Center Rotterdam, Rotterdam, The Netherlands; 6 Institute of Health Policy and Management, Erasmus University Rotterdam, Rotterdam, The Netherlands; London School of Hygiene & Tropical Medicine, UNITED KINGDOM

## Abstract

To eliminate and eradicate gambiense human African trypanosomiasis (HAT), maximizing the effectiveness of active case finding is of key importance. The progression of the epidemic is largely influenced by the planning of these operations. This paper introduces and analyzes five models for predicting HAT prevalence in a given village based on past observed prevalence levels and past screening activities in that village. Based on the quality of prevalence level predictions in 143 villages in Kwamouth (DRC), and based on the theoretical foundation underlying the models, we consider variants of the Logistic Model—a model inspired by the SIS epidemic model—to be most suitable for predicting HAT prevalence levels. Furthermore, we demonstrate the applicability of this model to predict the effects of planning policies for screening operations. Our analysis yields an analytical expression for the screening frequency required to reach eradication (zero prevalence) and a simple approach for determining the frequency required to reach elimination within a given time frame (one case per 10000). Furthermore, the model predictions suggest that annual screening is only expected to lead to eradication if at least half of the cases are detected during the screening rounds. This paper extends knowledge on control strategies for HAT and serves as a basis for further modeling and optimization studies.

## Author Summary

The primary strategy to fight gambiense human African trypanosomiasis (HAT) is to perform extensive population screening operations among endemic villages. Since the progression of the epidemic is largely influenced by the planning of these operations, it is crucial to develop adequate models on this relation and to employ these for the development of effective planning policies. We introduce and test five models that describe the expected development of the HAT prevalence in a given village based on historical information. Next, we demonstrate the applicability of one of these models to evaluate planning policies, presenting mathematical expressions for the relationship between participation in screening rounds, sensitivity of the diagnostic test, endemicity level in the village considered, and the screening frequency required to reach eradication (zero prevalence) or elimination (one case per 10000) within a given time-frame. Applying these expressions to the Kwamouth health zone (DRC) yields estimates of the maximum screening interval that leads to eradication, the expected time to elimination, and the case detection fraction needed to reach elimination within five years. This paper serves as a basis for further modeling and optimization studies.

## Introduction

Human African trypanosomiasis (HAT), also known as sleeping sickness, is a parasitic disease that is caused by two sub-species of the protozoa Trypanosoma brucei: Trypanosoma brucei gambiense (gambiense HAT) and Trypanosoma brucei rhodesiense (rhodiense HAT). The infection causing the disease is transmitted from person to person through the tsetse fly. It is estimated that there were 20000 cases in the year 2012 [1] and that 70 million people from 36 Sub-Saharan countries are at risk of HAT infection [2, 3].

Our work focuses on gambiense HAT, which represents 98% of all HAT cases [3]. Gambiense HAT, which we will refer to as “HAT” from now on, is a slowly progressing disease and is fatal if left untreated. In the first stage of the disease, symptoms are usually absent or non-specific [4]. The median duration of this stage is about 1.5 years [5]. By the time patients arrive at a healthcare provider, the disease has often progressed to the neurological phase, which causes severe health problems. In addition, this treatment delay increases the rate of transmission, since an infected patient is a potential source of infection for the tsetse fly [4, 6]. Therefore, active case finding and early treatment are key to the success of gambiense HAT control [7, 8].

The current case finding strategy uses mobile teams that travel from village to village to conduct exhaustive population screening [4, 8, 9]. For example, 35 mobile teams are active in the Democratic Republic of the Congo (DRC). Because this strategy has considerably reduced disease prevalence in several African countries [6, 10–12], the disease is no longer perceived as a major threat. Consequently, donors are now scaling down their financial commitments [8]. This, however, poses a serious risk to the control of HAT. The disease tends to re-emerge when screening activities are scaled down, bringing about the risk of a serious outbreak, as shown by an epidemic in the 1990s [4, 11, 13]. For example, the number of cases in 1998 is estimated to have exceeded 300000 [3].

In order to minimize the risk of re-emergence when resources are scaled down, and in order to eliminate and eradicate the disease, maximizing the *effectiveness* of the control programs is crucial. Mpanya et al. [9] suggest that the effectiveness of population screening is determined by (among others) the management and planning of the mobile teams. Planning decisions—which determine *which villages* to screen, and at what *time interval* to screen them—have a direct impact on the risk and the magnitude of an outbreak. Existing literature does not address these issues, as highlighted by the WHO [1], and a wide variety of screening intervals have been applied in different control programs [12, 14, 15]. To optimize the planning decisions, it is of key importance to be able to predict the evolution of the HAT prevalence level in the villages at risk. This allows decision makers to assess the relative effectiveness of a screening round in these villages and to prioritize the screening rounds to be performed.

However, practical tools for predicting HAT prevalence appear to be lacking. Existing models for HAT are mostly based on differential equations, describing the *rate of change* for the HAT prevalence level among humans and flies as a function of the prevalence levels among humans and flies (some models also include an animal reservoir) [16–22]. As the information needed to use such models—e.g., the number of tsetse flies in a village—is not available on the village level, using these models for prediction is impractical.

This paper therefore sets out to develop practical models describing and predicting the expected evolution of the HAT prevalence level in a given village, based on historical information on HAT cases and screening rounds in that village. The main difference with the models mentioned in the previous paragraph is that our models make no assumptions about the causal factors underlying the observed prevalence levels: the “inflow” of newly infected persons and the “outflow” of infected persons by cure or death. Instead, we just consider data on the net effect of these two processes—the evolution of the prevalence level—and fit five different models to this. To analyze the predictive performance of these models, we make use of a dataset describing screening operations and HAT cases in the Kwamouth district in the DRC for the period 2004–2013.

Furthermore, we use one of the models to analyze the fixed frequency screening policy, which assigns to each village a fixed time interval for consecutive screening rounds. Specifically, we investigate screening frequency requirements for reaching *elimination* and *eradication*. Here, eradication is defined as “letting the expected prevalence level go to zero in the long term”, and elimination is defined as “reaching an expected prevalence level of one case per 10000”.

Our paper thereby contributes to the branch of research on control strategies for HAT. Next, we list several other papers that are highly related to our work. The effectiveness of active case finding operations is analyzed by Robays et al. [23], who define “effectiveness” as the expected fraction of cases in a village which will eventually get cured as a result of a screening round in that village. The papers by Stone & Chitnis [16], Chalvet-Monfray et al. [20], and Artzrouni & Gouteux [24] introduce differential equation models to gain structural insights on the effectiveness of combinations of active case finding and vector control efforts and on the requirements for eradicating HAT. The effect of active case finding activities is modeled through a continuous “flow” of infected individuals into the susceptible compartment. Since we explicitly model the timing and the effects of a screening round, this is one of the main differences with our paper. Finally, Rock et al. [10] study the effectiveness of screening and treatment programs and the time to elimination using a multi-host simulation model. Their paper, however, considers the screening frequency as a given, whereas we consider the effects of changing this frequency. Furthermore, we propose models for predicting prevalence on a village level, whereas their model implicitly assumes all villages to be homogeneous.

## Materials and Methods

### Data

Our dataset consists of information on screening operations in the period 2004–2013 in the health zone Kwamouth in the province Bandundu. The raw data were cleaned up based on the rules described in S1 Text. The number of villages in the dataset equals 2324, and 143 of these villages were included in the data analysis based on three criteria: (1) the number of screening rounds recorded was at least two, (2) at least one case has been detected over the time horizon, and (3) at least one record of the number of people screened during the operation was available. The first condition is necessary to enable modeling the prevalence level observed in a given screening round as a function of past observed prevalence levels, and the third condition is necessary for estimating prevalence itself. We estimate the prevalence level in a village at the time of a screening round as the number of cases detected in that round over the number of people participating in that round. Furthermore, lacking population size data, we estimate the population of a village as the maximum number of people participating in a screening round reported for that village. Though our dataset also contains cases identified by the regular health system in between successive screening rounds, these do not yield (direct) estimates of prevalence levels in the corresponding villages, as required by the models proposed in the next section. We therefore focus on the active case finding data only.

**Fig 1:**
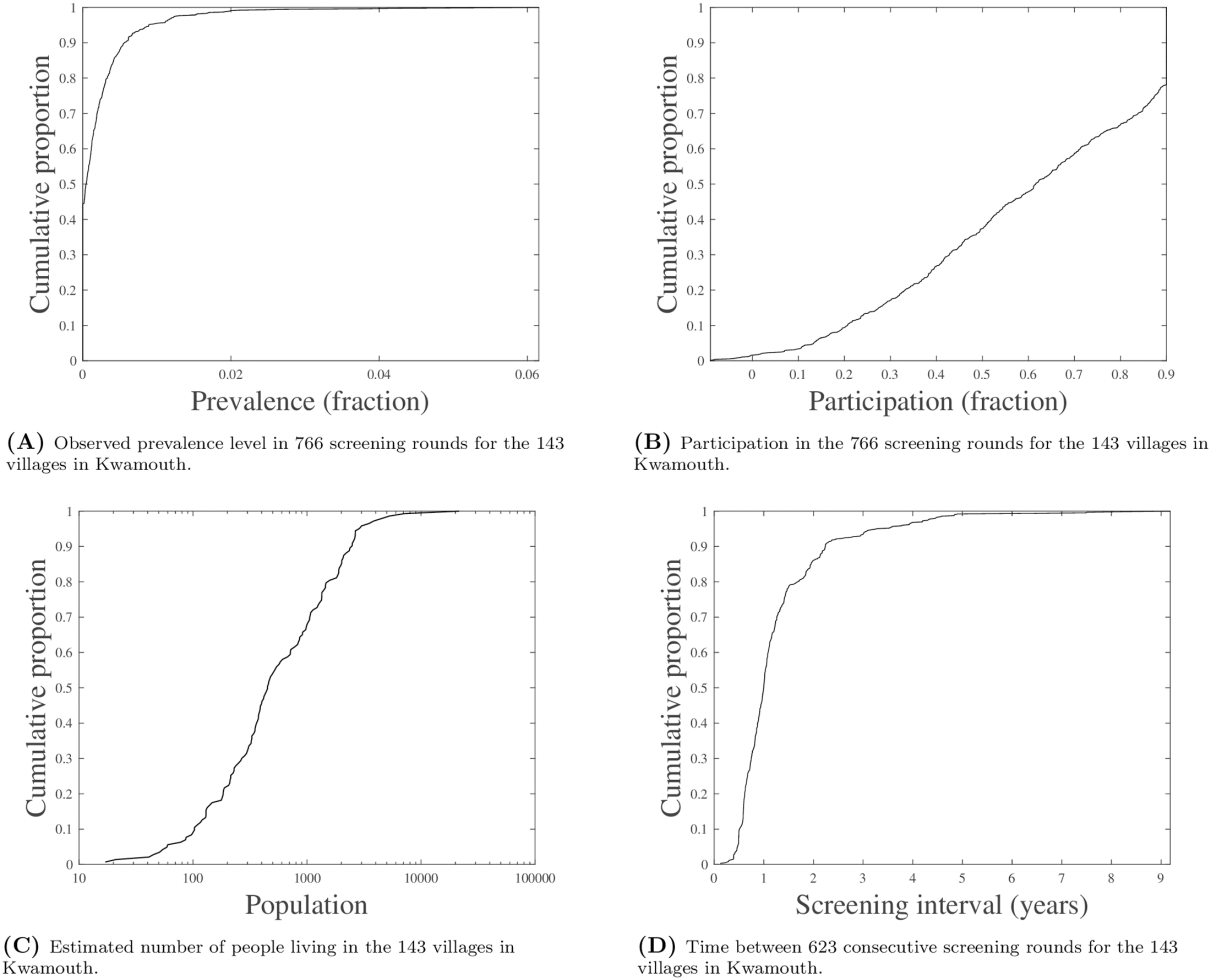
Descriptive statistics of the dataset.

The total number of screening rounds reported for the 143 villages included equals 766 (on average 5.4 per village). Fig 1 shows cumulative distributions of the observed prevalence level in these screening rounds (mean 0.0055, median 0.0011, standard deviation 0.0121), the time interval between each pair of consecutive screening rounds (mean 1.28, median 1.00, standard deviation 1.03), the estimated population for each village (mean 1073, median 450, standard deviation 2046), and the participation level in the screening rounds (mean 0.69, median 0.72, standard deviation 0.27). Note that the relatively large number of observations with a participation level of 100% is due to the method used to estimate the population sizes.

### Prediction Methods

Before we propose our prediction methods, we introduce some notations. A table of the most important notations used in this article can be found in S1 Table. Let **s**_*v*_ = {*s*_*v*1_, *s*_*v*2_, …} denote the vector of screening time intervals for village *v*, where *s*_*v*1_ denotes the time between the start of the time horizon and the first screening for this village, *s*_*v*2_ denotes the time between the first and the second screening, and so on. The time at which the *n*^*th*^ screening is performed is given by *S*_*vn*_ = ∑_*m*≤*n*_ *s*_*vm*_ and the participation fraction in this screening round is denoted by *p*_*vn*_. Parameter *N*_*v*_ represents the population size of village *v*. Furthermore, let **i**_*v*_ represent historical information on HAT cases in this village: the numbers of cases detected during past screening rounds. We model the *expected* prevalence level at time *t* in village *v* as a function *f*_*v*_(⋅) of **s**_*v*_, **i**_*v*_, and some parameters *β*: *f*_*v*_(*t*, **s**_*v*_, **i**_*v*_, *β*). Note that the expected prevalence level is a *latent*, i.e. unobserved, variable, and that the observed prevalence level, *x*_*v*_(*t*), generally deviates from the expected value. We measure prevalence levels *f*_*v*_(⋅) and *x*_*v*_(⋅) as *fractions* and represent the difference between the expected and observed prevalence level in village *v* by the random variable *ε*_*v*_:

$$x_{v}\left(t\right)=f_{v}\left(t,s_{v},i_{v},\beta \right)+\varepsilon_{v}$$ (1)

Time series models such as discrete time ARMA, ARIMA or ARIMAX models seem to be the most popular methods for predicting prevalence (or incidence) (see e.g. [25, 26]). These models describe the prevalence level at time *t* as a linear function of the prevalence levels at time *t* − 1, *t* − 2, … and (optionally) some other variables. Their applicability in our context is however limited. Discrete time models require estimates of the prevalence level at each time unit (e.g., each month), whereas information to estimate the HAT prevalence level is available only at moments at which a screening round is performed. Namely, many HAT patients are not detected by the regular health system, particularly if they are in the first stage of the disease [8].

The class of continuous time models is much more suitable for analyzing data observed at irregularly spaced times. These models assume that the variable of interest, *f*_*v*_(*t*, **s**_*v*_, **i**_*v*_, *β*), follows a continuous process, defining its value at each *t* > 0. The next subsections propose five continuous time models for predicting HAT prevalence levels. We again note that models describing the causal processes determining the observed prevalence levels in detail (e.g., by explicitly modelling disease incidence, passive case finding, death and cure) may be most intuitive, but require data that are not available on a village level. Therefore, to safeguard their relevance for practical application, the variables we include are only those that are available on a large scale. This does not imply that our models neglect the causal processes. Instead, they are to some extent accounted for in an implicit way by fitting the models to the observed prevalence levels.

Data that are typically available at village level are numbers of HAT cases found during screening rounds and the times of these screening rounds. For a given village, the first yields estimates of *past prevalence levels*, and the latter yield the *time intervals* between past screening rounds. We hypothesize that the current expected prevalence level at time *t* is related to past prevalence levels, past screening intervals, and in particular the time since the last screening round, which we denote by $\delta_{v}^{-}\left(t\right)=\min_{n}\left\{t-S_{vn}|S_{vn}\leq t\right\}$. Hence, we include (functions of) these variables in our models.

Linear regression models are very widely used in the world of forecasting (see e.g. [27]). Major advantages of these models are that they are easy to understand, to implement, to fit, and to analyze. Therefore, the first model we introduce is a linear model (model 1), which also serves as a benchmark for our more advanced models. This model describes the expected HAT prevalence in a given village as a function of the time since the last screening and past prevalence levels. Such linear model is, however, very vulnerable to a typical structure present in active case finding datasets. High past prevalence levels tend to increase the priority of screening a village, causing the time intervals between screening rounds to decrease. As a result, $\delta_{v}^{-}\left(t\right)$ is a highly “endogenous” variable. More formally, external variables (past prevalence levels) are correlated with both the dependent variable (*f*_*v*_(*t*)) and the independent variable ($\delta_{v}^{-}\left(t\right)$), which makes it hard to quantify the (causal) relation between them. In response to this, we present four alternative models. Model 2 is a fixed effects model, which adds a dummy-variable for each village to the initial model. Model 3 is a (non-linear) exponential growth and decay model which is inspired by the SIS epidemic model. This model is being used extensively for modeling epidemics that are characterized by an initial phase in which the number of infected individuals grows exponentially, and a second phase in which this number levels off to a time-invariant *carrying capacity*. We refer to model 3 as the logistic model with a constant carrying capacity. Finally, model 4 is a less data dependent version of model 3 and model 5 is variant of model 3 in which the carrying capacity is allowed to vary over time.

#### Model 1: Linear Model

Let *x*_*v*_(*S*_*vn*_) and ${\bar{x}}_{v}\left(S_{vn}\right)$ represent the observed prevalence level exactly at the *n*^*th*^ screening round in village *v* and the average observed prevalence level in the *three years prior to* the *n*^*th*^ screening round in village *v*, respectively. (For screening rounds in the first *T* < 3 years of our dataset, ${\bar{x}}_{v}\left(S_{vn}\right)$ represents the average prevalence levels in these *T* years). The linear model (LM) describes the expected prevalence level at time *t*, $f_{v}\left(S_{vn}+\delta_{v}^{-}\left(t\right)\right)$ as a function of the time since the last screening round in village *v* before time *t*, and past observed prevalence levels in that village:

$$\begin{array}{ll} x_{v}(S_{vn}+\delta_{v}^{-}(t)) & =f_{v}(S_{vn}+\delta_{v}^{-}(t))+\varepsilon_{v}\\ & =\beta_{1}{\bar{x}}_{v}(S_{vn})+\beta_{2}x_{v}(S_{vn})+\beta_{3}\sqrt{\delta_{v}^{-}(t)}+\beta_{4}\delta_{v}^{-}(t)+\beta_{5}{\bar{x}}_{v}(S_{vn})\sqrt{\delta_{v}^{-}(t)}+\beta_{6}x_{v}(S_{vn})\sqrt{\delta_{v}^{-}(t)}+\beta_{7}{\bar{x}}_{v}(S_{vn})\delta_{v}^{-}(t)+\beta_{8}x_{v}(S_{vn})\delta_{v}^{-}(t)+\varepsilon_{v} \end{array}$$ (2)

We include the term $\sqrt{\delta_{v}^{-}\left(t\right)}$ into this model to incorporate the non-linear nature that characterizes epidemics: the (increase in the) number of cases typically levels off after an initial phase of fast growth. Furthermore, we include the cross terms based on the hypothesis that the prevalence level increases faster over time for villages which have higher past prevalence levels.

#### Model 2: Fixed Effects Model

Datasets about the HAT epidemic, such as the data set used in this study, show much similarity to *panel data* in the sense that both our data and panel data consist of observations at multiple points in time for multiple units (i.e., villages). The causality problem mentioned in the introduction of this section can be regarded as the problem that differences between units explain part of the regressors. A common way to deal with differences between units in panel data is to assume that there is a unit-specific, time-invariant *fixed effect* that contributes to the dependent variable. This is modeled by adding a constant for each unit to the regression model. Thereby, the model relates deviations from the fixed effect to variations in the explanatory variables. In line with this, we consider the following regression model, which we denote by the fixed effects model (FEM):

$$\begin{array}{l} \begin{array}{ll} x_{v}(S_{vn}+\delta_{v}^{-}(t)) & =f_{v}(S_{vn}+\delta_{v}^{-}(t))+\varepsilon_{v}\\ & =\alpha_{v}+\beta_{1}{\bar{x}}_{v}(S_{vn})+\beta_{2}x_{v}(S_{vn})+\beta_{3}\sqrt{\delta_{v}^{-}(t)}+\beta_{4}\delta_{v}^{-}(t)+\beta_{5}{\bar{x}}_{v}(S_{vn})\sqrt{\delta_{v}^{-}(t)}+\beta_{6}x_{v}(S_{vn})\sqrt{\delta_{v}^{-}(t)}+\beta_{7}{\bar{x}}_{v}(S_{vn})\delta_{v}^{-}(t)+\beta_{8}x_{v}(S_{vn})\delta_{v}^{-}(t)+\varepsilon_{v} \end{array} \end{array}$$ (3)

Here, *α*_*v*_ represents the fixed effect for village *v*. Note that the term *α*_*v*_ forms the only difference between the FEM and the linear model.

#### Model 3: Logistic Model with a Constant Carrying Capacity

Literature suggests that the development of the expected fraction of individuals infected with HAT grows exponentially in a first phase and that this fraction levels off to (or oscillates around and converges to) an equilibrium prevalence level in a second phase [21, 22]. We refer to the equilibrium prevalence level as the *carrying capacity*. A simple epidemic model that incorporates these effects is the SIS model, which describes the evolution of the expected prevalence level in village *v* by means of the following differential equation:

$$\frac{df_{v}\left(t\right)}{dt}=\kappa \cdot f_{v}\left(t\right)\left(1-\frac{f_{v}\left(t\right)}{K_{v}}\right)$$ (4)

Here, as in the previous section, *f*_*v*_(*t*) denotes the expected fraction of people in population *v* who are infected at time *t*, *K*_*v*_ represents the carrying capacity of this village, and *κ* represents a parameter indicating the speed of convergence to the epidemic equilibrium. When the epidemic is in its initial phase, the growth rate of *f*_*v*_(*t*) is approximately *κ* ⋅ *f*_*v*_(*t*), whereas this rate goes to 0 when *f*_*v*_(*t*) approaches the carrying capacity *K*_*v*_. We propose to use the explicit solution to this differential equation to model the development of the expected HAT prevalence level in village *v*. This solution is obtained by multiplying both sides of Eq (4) by *dt*, taking the integral on both sides, and regarding the previous screening (i.e., the one performed at time *S*_*vn*_) as the beginning of the time horizon:

$$f_{v}\left(S_{vn}+\delta_{v}^{-}\left(t\right)\right)=\frac{K_{v}}{1+A_{vn}\cdot e^{-\kappa \cdot \delta_{v}^{-}\left(t\right)}}$$ (5)

*A*_*vn*_ can be interpreted as a parameter determining the expected prevalence level *immediately after* the *n*^*th*^ screening round in village *v*: $A_{vn}=\frac{K_{v}}{f_{v}\left(S_{vn}^{+}\right)}-1$, where $S_{vn}^{+}$ denotes this moment.

We fit the parameters in function (5) using the dataset described in the previous section. To keep the model simple, we assume that *κ* is constant over time and over villages (i.e., this parameter is an intrinsic property of the epidemic). Furthermore, we specify an interval of realistic values for *κ* based on the following reasoning. Note that *κ* represents the expected number of new infections per infected person per time unit in a population that is almost completely susceptible: *f*_*v*_(*t*) → 0. Now let *r* represent the yearly removal rate for HAT—the rate of progression to death or cure—and let *R*_0_ denote the basic reproduction number for HAT—the average number of secondary cases induced by one case in an otherwise susceptible population. Since the expected disease duration of this one case is $\frac{1}{r}$ years, the average rate at which secondary infections are induced by this one case equals $\frac{R_{0}}{\frac{1}{r}}=r\cdot R_{0}$ per year. Under the assumption that this rate is constant over the disease duration, this implies that *κ* = *r* ⋅ *R*_0_. Since several analyses suggest that the value of *R*_0_ is generally close to 1 in endemic regions [10, 17, 29], we hypothesize that *R*_0_ ∈ [1.0, 1.5]. Furthermore, 95% confidence intervals for the stage 1 and stage 2 disease duration, as presented by Checchi et al. [5], suggest that *r* lies in the interval [0.22, 0.52]. Based on these ranges we estimate that *κ* lies in the interval [0.2, 0.8]. We note that one could try to estimate village-specific or focus-specific ranges for *R*_0_ (and hence for *κ*) using the next-generation matrix method, as proposed by [28]. Yet, as the corresponding data requirements are relatively large, we deem this to be practically unsuitable.

Furthermore, we hypothesize that *K*_*v*_ is highly related to past prevalence levels and past screening activities in village *v*. Higher prevalence levels indicate a higher carrying capacity. Furthermore, if two villages had equal prevalence levels, but if village 1 has been screened more frequently than village 2, this may indicate that the carrying capacity in village 1 is higher. In accordance with these hypotheses, we estimate *K*_*v*_ as:

$$K_{v}=\beta_{1}+\beta_{2}{\tilde{\mu }}_{v}{\tilde{x}}_{v}$$ (6)

Here, ${\tilde{x}}_{v}$ and ${\tilde{\mu }}_{v}$ denote the observed average prevalence level in village *v* during five consecutive years and the average screening frequency (screening rounds per year) in this village during these years, respectively. We take the last five years of data in our estimation sample (as we discuss in the next section, we split up the dataset in an estimation sample and a prediction sample). If only *T* < 5 years of data are available, we base ${\tilde{x}}_{v}$ and ${\tilde{\mu }}_{v}$ on these *T* years. Note that variables ${\tilde{x}}_{v}$ and ${\tilde{\mu }}_{v}$ are based on historical information and hence that these variables (and hence the estimated carrying capacity) are not affected by current screening frequency decisions. Furthermore, we note that these variables should be based on the same period of five years, since *combined information* on the screening efforts and resulting prevalence levels provides insight into the epidemic potential in a village.

The estimate of *K*_*v*_ provides one of the two inputs for determining *A*_*vn*_. The second input is the expected prevalence level in village *v* at time $S_{vn}^{+}$ (i.e., immediately after the *n*^*th*^ screening round). Under the assumption that infected individuals who are detected during a screening round are immediately “removed”, the only people infected at time $S_{vn}^{+}$ are those who did not participate in the screening round and those not detected by the diagnostic test. This suggests the following relation between the expected prevalence after and the expected prevalence before the *n*^*th*^ screening round in village *v*:

$$f_{v}\left(S_{vn}^{+}\right)=\left(1-p_{vn}\cdot s\right)f_{v}\left(S_{vn}^{-}\right)$$ (7)

Here, *p*_*vn*_ and *s* denote the participation in the *n*^*th*^ screening in village *v* and the sensitivity of the diagnostic test, and $S_{vn}^{-}$ denotes the moment right *before* the infected individuals were removed. We assume that *s* = 0.925 based on the review by Brun et al. [4], and obtain the following estimator for *A*_*vn*_:

$$A_{vn}=\frac{K_{v}}{f_{v}\left(S_{vn}^{+}\right)}-1=\frac{\beta_{1}+\beta_{2}{\tilde{\mu }}_{v}{\tilde{x}}_{v}}{\left(1-p_{vn}\cdot s\right)f_{v}\left(S_{vn}^{-}\right)}-1$$ (8)

The model requires an assumption about the (unobserved) expected prevalence level at the beginning of the time horizon (i.e., at 01-01-2004). The only information we have about this level is that it is lower than the expected prevalence level at the first screening round. Furthermore, under the realistic assumption that the epidemic is in its “convex part”, the expected prevalence level will have steeply increased between 01-01-2004 and the first screening round, suggesting that there is a substantial difference between the two. Therefore, taking ${\tilde{x}}_{v}$ as an estimate of the expected prevalence level at the first screening round and lacking further information, we choose to estimate *f*_*v*_(0) as half the average *observed* prevalence level during five years: $0.5{\tilde{x}}_{v}$. The section Comparison of Models discusses the sensitivity of our results with respect to this choice.

Substituting Eqs (6) and (8) into the definition of the prevalence level Eq (5) we obtain the following model, which we refer to as the Logistic Model with a Constant Carrying Capacity (LMCCC), and which we fit by estimating the parameters *κ*, *β*_1_, and *β*_2_:

$$\begin{array}{ll} x_{v}(S_{vn}+\delta_{v}^{-}(t)) & =f_{v}(S_{vn}+\delta_{v}^{-}(t))+\varepsilon_{v}\\ & =\frac{\beta_{1}+\beta_{2}{\tilde{\mu }}_{v}{\tilde{x}}_{v}}{1+\left(\frac{\beta_{1}+\beta_{2}{\tilde{\mu }}_{v}{\tilde{x}}_{v}}{\left(1-p_{vn}\cdot s)f_{v}(S_{vn}^{-}\right)}-1\right)\cdot e^{-\kappa \cdot \delta_{v}^{-}(t)}}+\varepsilon_{v} \end{array}$$ (9)

Note that the expected prevalence level before the *n*^*th*^ screening round, $f_{v}\left(S_{vn}^{-}\right)$, depends on the estimated values of *κ*, *β*_1_, and *β*_2_. In fact, by substituting its definition, Eq (9) can be written in the following form (see S2 Text):

$$x_{v}\left(S_{vn}+\delta_{v}^{-}\left(t\right)\right)=\frac{\beta_{1}+\beta_{2}{\tilde{\mu }}_{v}{\tilde{x}}_{v}}{1+\sum_{i\leq n}a_{vi}e^{-\kappa \cdot \delta_{vi}}}+\varepsilon_{v}$$ (10)

Here, *a*_*vi*_ and *δ*_*vi*_ denote some nonnegative constants.

#### Model 4: Restricted Logistic Model with a Constant Carrying Capacity

Estimates of historical prevalence levels ${\tilde{x}}_{v}$ are available on a very large scale. The national HAT control programs are a main source of these data, as they keep track of all HAT cases found. Alternatively, modeling studies have yielded estimates of the incidence levels in Sub-Saharan Africa at the level of detail of 1 km^2^ (see [2, 30]), which can be transformed into corresponding estimates of prevalence levels. Data to measure ${\tilde{\mu }}_{v}$, the historical screening frequency in village *v*, are however scarcer in general, as gathering these data brings about a significant administrative burden. It is therefore relevant to investigate the predictive performance of a model that only uses ${\tilde{x}}_{v}$ to estimate *K*_*v*_ instead of ${\tilde{\mu }}_{v}{\tilde{x}}_{v}$. We do so by fitting the following model, which we refer to as the restricted Logistic Model with a Constant Carrying Capacity (rLMCCC):

$$x_{v}\left(S_{vn}+\delta_{v}^{-}\left(t\right)\right)=\frac{\beta_{1}+\beta_{2}{\tilde{x}}_{v}}{1+\left(\frac{\beta_{1}+\beta_{2}{\tilde{x}}_{v}}{\left(1-p_{vn}\cdot s\right)f_{v}\left(S_{vn}^{-}\right)}-1\right)\cdot e^{-\kappa \cdot \delta_{v}^{-}\left(t\right)}}+\varepsilon_{v}$$ (11)

#### Model 5: Logistic Model with a Varying Carrying Capacity

The models presented in the previous subsections implicitly assume that the carrying capacity (i.e., the upper bound on the expected prevalence level) remains the same over time. However, due to possible changes in conditions that affect the disease dynamics—such as vegetation, the number of flies and humans in and around a village, passive case finding activities—it is realistic to assume that the carrying capacity may vary over time. Furthermore, the epidemic potential may also vary due to variations in susceptibility to or tolerance of HAT infection, as argued by Welburn et al. [31]. Based on this assumption, we propose a Logistic Model with a Varying Carrying Capacity (LMVCC):

$$x_{v}\left(S_{vn}+\delta_{v}^{-}\left(t\right)\right)=\frac{\beta_{1}+\beta_{2}{\bar{\mu }}_{v}\left(S_{vn}\right){\bar{x}}_{v}\left(S_{vn}\right)}{1+\left(\frac{\beta_{1}+\beta_{2}{\bar{\mu }}_{v}\left(S_{vn}\right){\bar{x}}_{v}\left(S_{vn}\right)}{\left(1-p_{vn}\cdot s\right)f_{v}\left(S_{vn}^{-}\right)}-1\right)\cdot e^{-\kappa \cdot \delta_{v}^{-}\left(t\right)}}+\varepsilon_{v}$$ (12)

The only change with respect to the logistic model with a constant carrying capacity is that ${\tilde{x}}_{v}$ and ${\tilde{\mu }}_{v}$ are replaced by ${\bar{x}}_{v}\left(S_{vn}\right)$ and ${\bar{\mu }}_{v}\left(S_{vn}\right)$, the average observed prevalence level in and the screening frequency for village *v* in the *three years prior to* the *n*^*th*^ screening round in that village, respectively.

### Model Fitting

As HAT prevalence levels are very low, the variance of these levels is high, which enhances the chance that there are significant outliers among the observations. For example, no cases were detected in three out of four screening rounds performed in a village of 122 people, whereas five cases were detected in the 4^*th*^ round. This implies two things. First, observed prevalence levels will generally deviate significantly from expected prevalence levels. Second, we need to choose a technique for estimating the model coefficients that is robust with respect to outliers. Instead of Least Squares (LS) regression, one of the most commonly applied model fitting methods, we therefore use Least Absolute Deviations (LAD) regression to fit the model parameters, which is known to be relatively insensitive to outlying observations [32]. An alternative technique would be to use a maximum likelihood estimation (MLE) approach based on a heavy-tailed probability distribution for the observed prevalence levels. In S2 Table, we show the results obtained when assuming a Poisson, Beta-Binomial, or Negative Binomial distribution. Each of the MLE approaches is, however, clearly outperformed by the LAD regression approach.

The variance of the observed prevalence level strongly depends on the sample size. For example, under the assumption of an independent infection probability for each person, the variance is inversely proportional to the sample size. We therefore weight the fitting deviation *e*_*vn*_ = *f*_*v*_(*S*_*vn*_) − *x*_*v*_(*S*_*vn*_) for observation *n* for village *v* by weight $w_{vn}=\sqrt{N_{v}\cdot p_{vn}}$, yielding the following weighted LAD regression problem:

$$\min_{\beta }S_{abs}\left(\beta \right)=\sum_{\left(v,n\right)}w_{vn}\left|e_{vn}\right|$$ (13)

To deal with the risk of overfitting, we select the variables to be included in the models by means of a backward elimination method. This method initially includes all variables in the model and iteratively removes the least significant variable (if its *p*-value > 0.10) and estimates the model with the remaining variables. The algorithm stops as soon as all remaining variables are significant or if only one variable is left. We enforce that *α*_*v*_ and *κ* cannot be removed by the backward elimination method so as to preserve essential elements of the corresponding models. Hence, only parameters *β*_1_-*β*_8_ in models 1 and 2, and parameters *β*_1_-*β*_2_ in models 3–5 could be removed.

Finally, to test the predictive performance of the models, we split the data in an estimation sample (which we use for fitting the model) and a prediction sample. Specifically, for each of the 143 villages, we include the last screening round in the prediction sample, and include the others in the estimation sample. Next, we measure performance based on the mean of the prediction errors $ME=\frac{\sum_{v}e_{\hat{vn}}}{\left|V\right|}$, indicating whether the predictions obtained by the model are *biased*, and based on two indicators for the amount of *explained variation* in the prevalence levels: the mean absolute error, $MAE=\frac{\sum_{v}\left|e_{\hat{vn}}\right|}{\left|V\right|}$ and the mean relative error, $MRE=\sum_{v}\frac{|e_{\hat{vn}}|}{\sum_{v}x_{v}\left(S_{\hat{vn}}\right)}$. Here, the index combination $\hat{vn}$ indicates the last screening round for village *v*. The intuition behind the measures of explained variation is that they equal 0 if the predicted prevalence levels are exactly equal to the observed prevalence levels (i.e., the model perfectly explains the variation in the observed prevalence levels) and that their value increases when the absolute difference between predicted and observed levels increases (i.e. when the model explains less variation in observed prevalence levels). We use Matlab R2015b for the implementation of our methods.

## Results

### Fitted Models

Table 1 presents the coefficient estimates for the variables of the five presented models. The results for models 1 and 2 are very similar. Seven of the eight variables are identified as being non-significant by the backward elimination algorithm: the interaction terms, the long term prevalence level, the time since the last screening round, and the square root of the time since the last screening round. The resulting model provides a clear prediction method: the expected prevalence equals 24.5% of the prevalence level observed at the previous screening round (note, if this level was 0.0%, the estimated expected prevalence remains 0.0%) according to model 1, and equals 14.7% of this prevalence level plus a constant fraction *α*_*v*_ according to model 2. Hence, this model predicts that, in the absence of screening activities, the expected prevalence remains the same over time.

**Table 1:** Estimated weighted LAD regression models.

Variable	Coefficient name	Coefficient value	Std error	P-value
Model 1: Linear Model				
*x*_*v*_(*S*_*vn*_)	*β*_2_	0.245	0.035	0.000
Model 2: Fixed Effects Model*				
*x*_*v*_(*S*_*vn*_)	*β*_2_	0.147	0.055	0.004
Model 3: Logistic Model with a Constant Carrying Capacity				
${\tilde{\mu }}_{v}{\tilde{x}}_{v}$	*β*_2_	1.763	0.409	0.000
$\delta_{v}^{-}\left(t\right)$	*κ*	0.800	0.086	0.000
Model 4: Restricted Logistic Model with a Constant Carrying Capacity				
${\tilde{x}}_{v}$	*β*_2_	1.234	2.210	0.288
$\delta_{v}^{-}\left(t\right)$	*κ*	0.800	0.130	0.000
Model 5: Logistic Model with a Varying Carrying Capacity				
Constant	*β*_1_	0.004	0.001	0.002
${\bar{\mu }}_{v}\left(S_{vn}\right){\bar{x}}_{v}\left(S_{vn}\right)$	*β*_2_	0.330	0.157	0.018
$\delta_{v}^{-}\left(t\right)$	*κ*	0.800	0.123	0.000

* For sake of conciseness, the estimates of *α*_*v*_ are not given here.

**Fig 2:**
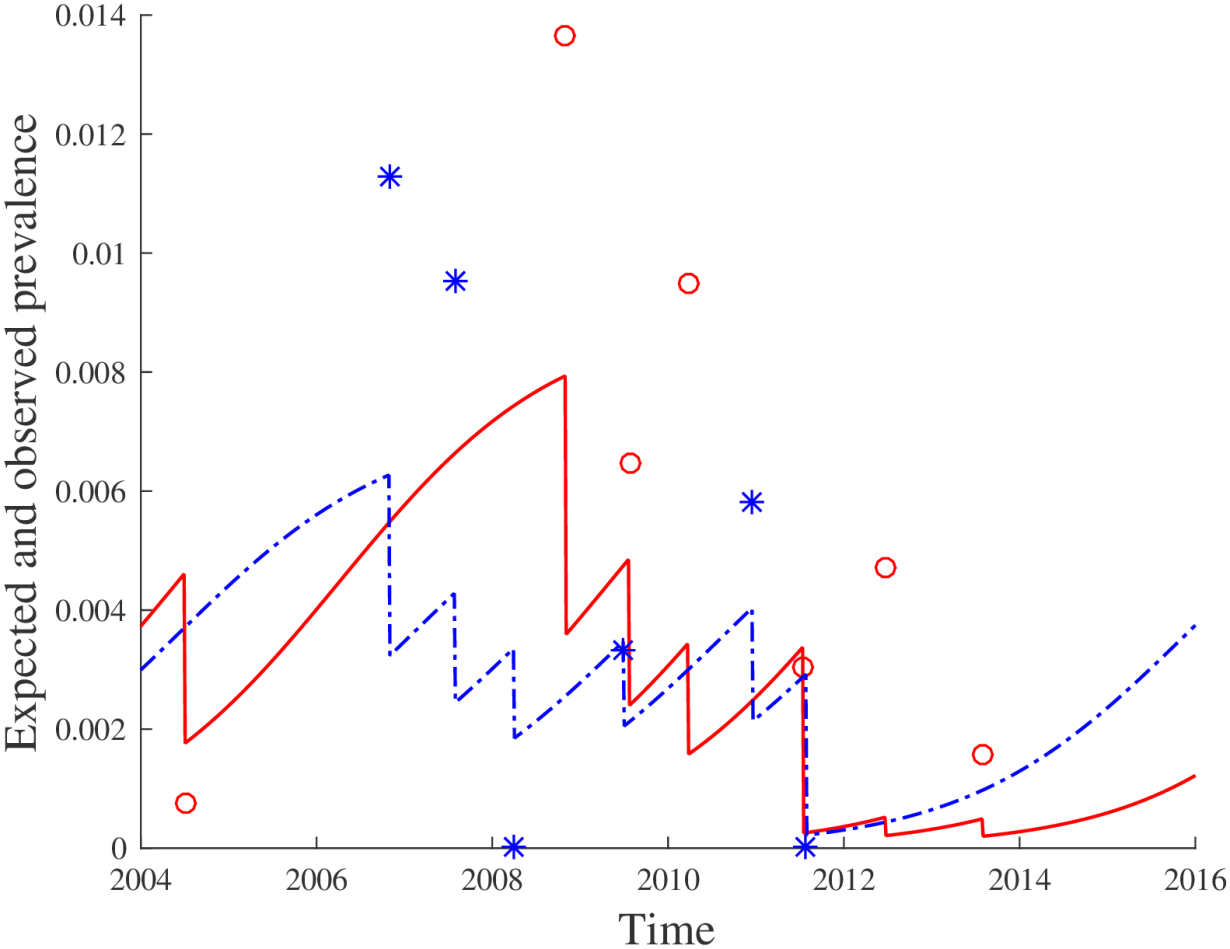
Evolution of the expected prevalence level over time for two villages according to the LMCCC model (lines) versus the observed prevalence levels (circles and stars). Village 1: red solid line vs. red circles. Village 2: blue dashed line vs. blue stars.

**Fig 3:**
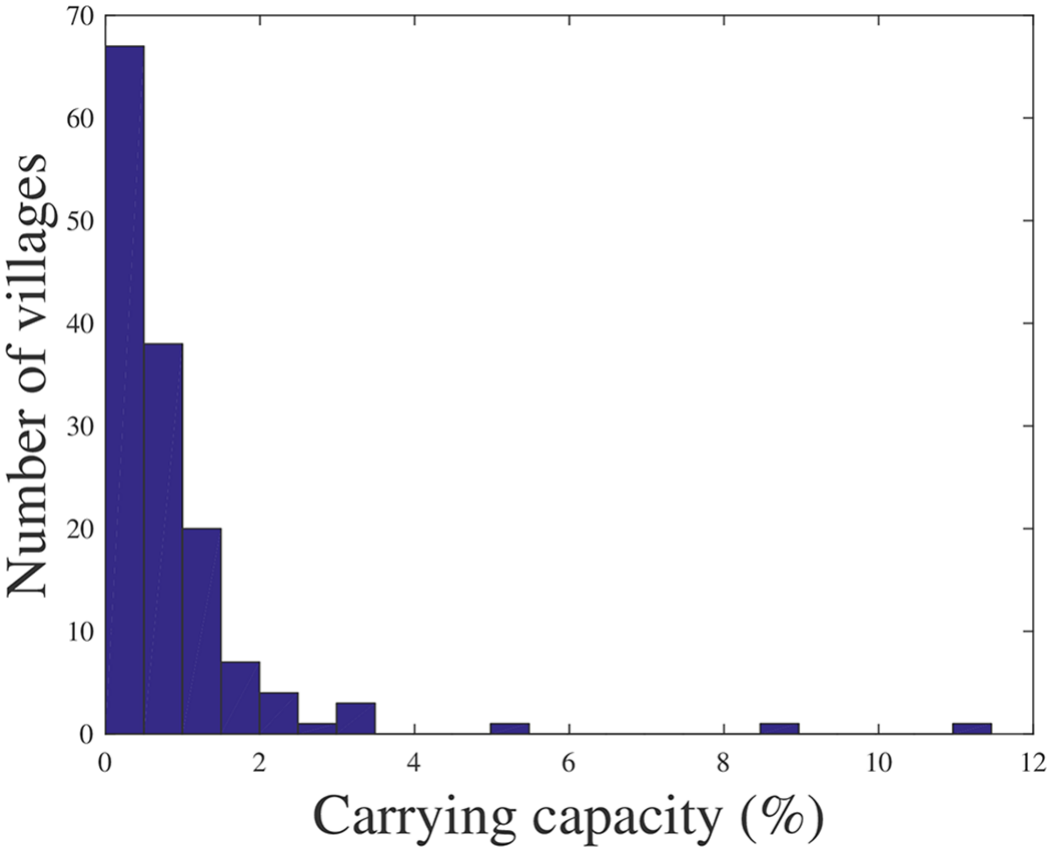
Histogram of estimated carrying capacities for the 143 villages in Kwamouth, as estimated by the LMCCC model. Measured as a percentage of the population.

The fitted models 3, 4, and 5 reveal a clear and intuitive relationship between screening frequency, prevalence, and carrying capacity: a larger historical prevalence indicates a higher carrying capacity, and facing an equal historical prevalence for a higher historical screening frequency indicates a higher carrying capacity. The constant term has been identified as non-significant for models 3 and 4 and as significant for model 5. To illustrate the typical output of models 3 and 4, Fig 2 shows the development of the expected prevalence levels for two villages over time (the lines), as well as the observed prevalence levels (stars and circles). Furthermore, Fig 3 depicts the carrying capacities for the 143 villages in Kwamouth, as estimated by the LMCCC model. Though data to validate these estimates are lacking, we note that they are in the same order of magnitude as prevalence levels found during screening rounds. The latter are usually between 1% and 5% in high or very high transmission areas, and exceed 10% in some extreme cases [33, 34].

### Comparison of Models

**Fig 4:**
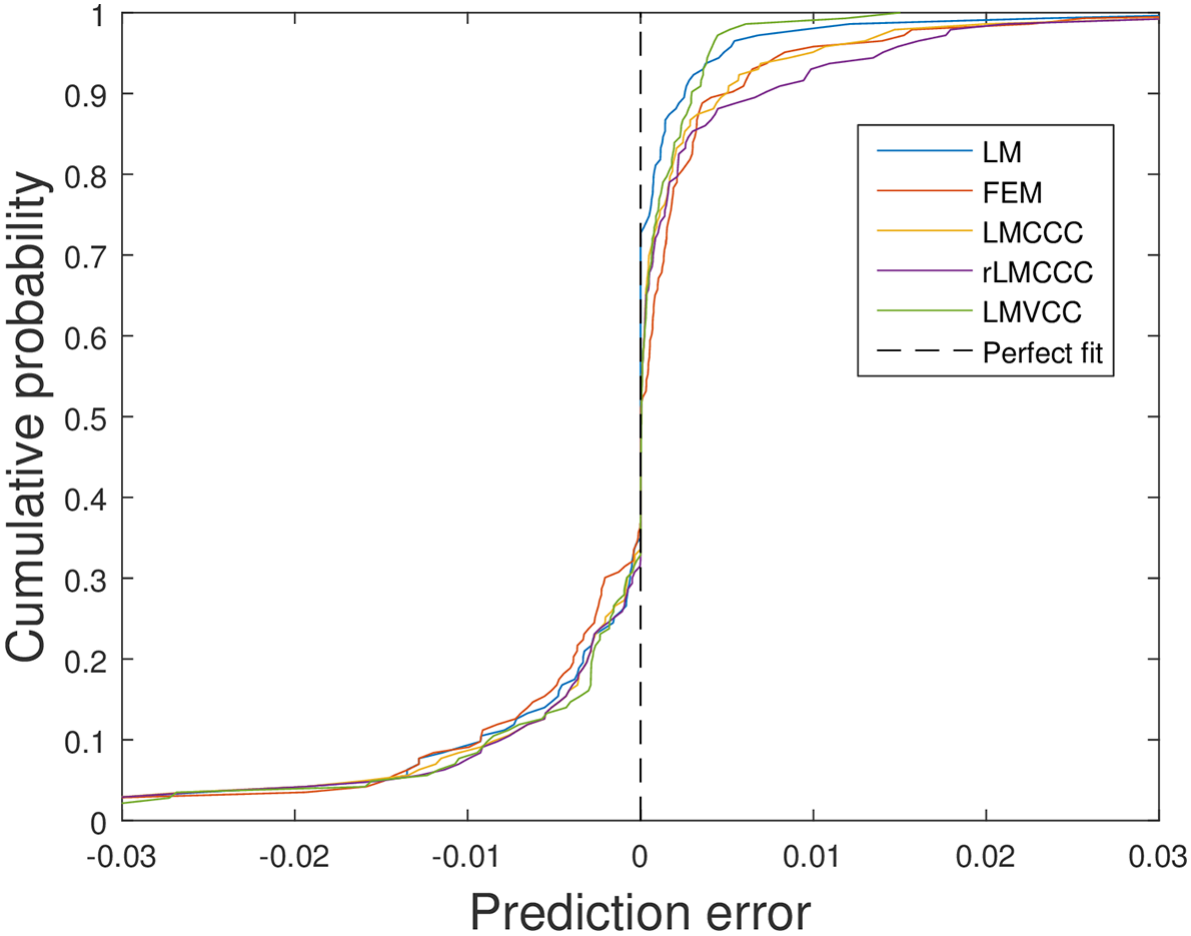
Prediction errors (predicted prevalence—observed prevalence) for the 143 observations in the prediction sample.

As mentioned in Section Model Fitting, we measure the predictive performance of the different models in terms of the prediction bias and in terms of the amount of variation explained. Table 2 contains the values of the different indicators for each of the models, and Fig 4 and S1 Fig. compare the prediction errors produced by the different models. These prompt several interesting observations.

**Table 2:** Comparison of the predictive performance of the five models in terms of mean errors (*ME*), mean absolute errors (*MAE*), and mean relative errors (*MRE*). The best indicator values are in bold.

	*ME*	*MAE*	*MRE*
Model 1: LM	−0.00186	0.00401	1.26
Model 2: FEM	−0.00105	0.00507	1.60
Model 3: LMCCC	−0.00094	0.00476	1.50
Model 4: rLMCCC	**0.00047**	0.00600	1.89
Model 5: LMVCC	−0.00176	**0.00363**	**1.14**

First, the prediction bias ranges from 0.47/1000 (rLMCCC model) to -1.86/1000 (LM model). Given that the average observed prevalence in the 766 screening rounds in our dataset equals 5.5/1000, we consider the biases of the LM model and the LMVCC model as quite substantial. Yet, this may be very well explained by the highly variable character of the HAT epidemic. A small number of outbreaks may substantially shift the average observed prevalence level. For example, without the four most negative prediction errors, the prediction bias for the LM model would be only -0.69/1000.

Second, the LM model performs relatively well in terms of explained variation. Yet, we see two vulnerabilities of this model: (1) as discussed before, this model is likely to be hampered by endogeneity, inducing a potential bias in the coefficient estimates, and (2) the variation in the screening intervals is relatively small for the villages with the highest endemicity levels in our sample, as these villages are screened almost every year. When there is little variation in $\delta_{v}^{-}\left(t\right)$, the true effects of variations might not become visible. These two fundamental vulnerabilities may very well explain why (a function of) $\delta_{v}^{-}\left(t\right)$ has not been identified as significant for the LM model. As a result, this model unrealistically predicts that the value of the expected prevalence level remains the same over time in the absence of screening activities, contrasting with vast historical evidence.

The same vulnerabilities apply to the FEM model, which also provides a counter-intuitive relation between the expected prevalence and $\delta_{v}^{-}\left(t\right)$. On top of that, its predictive power is relatively low, which could be explained by the fact that, for many villages, there is insufficient data to estimate the fixed effect accurately.

As variants of the logistic model already fix the structure of the relationship between $\delta_{v}^{-}\left(t\right)$ and $f_{v}\left(S_{vn}+\delta_{v}^{-}\left(t\right)\right)$ based on epidemiological insights, these models do not suffer from the vulnerabilities mentioned above. We therefore consider these models to have most potential for accurately predicting HAT prevalence levels in general (i.e., in any region and for any time horizon). Among the three logistic model variants, model 3 (LMCCC) performs reasonably well in terms of both criteria. Model 5 (LMVCC) has a substantial prediction bias, but performs best in terms of explained variation, as can be seen in Fig 4 (its performance is closest to the “perfect fit”). Though model 4 (rLMCCC) performs best in terms of prediction bias, it performs very weakly in terms of explained variation.

Hence, among the logistic model variants, there is no clear winner when both criteria are assigned equal importance. For planning decisions, however, we consider model 5 to be most suitable, followed by model 3. The reason is that, in contrast with prediction bias, explained variation indicates the ability to identify differences in expected prevalence levels between villages, as required for effective planning decisions. Hence, identifying an effective *prioritization* of the different villages will be more important than obtaining unbiased estimates of the resulting *prevalence* levels.

The sensitivity level *s* is known to differ between regions [35]. Furthermore, the population size of a village had to be estimated, which induces a potential bias in the participation level estimates. These issues beg to question the robustness of our results on the logistic model variants (note, models 1 and 2 are not affected by this as these do not use these parameters). S3 Table shows the results of a sensitivity analysis, which largely confirm our findings. In all scenario’s analyzed, model 5 remains best in terms of explained variation, followed by model 3, and models 3 and 4 outperform model 5 in terms of prediction bias. Another assumption that questions the robustness of our results is the one about the expected prevalence level at the beginning of the time horizon (i.e., at 01-01-2004). S4 Table provides the results of a sensitivity analysis on this assumption. Again our main findings remain the same.

### Analysis of the Fixed Frequency Screening Policy

In the previous section we argue that, among the models analyzed in this paper, variants of the logistic model have most potential for accurately predicting HAT prevalence levels in general. In this section we demonstrate the applicability of one of these model variants to analyze the effectiveness of screening operations. In particular, since information on the development of the carrying capacities is lacking, as required by the LMVCC model, and since we consider the predictive performance of model 3 superior to that of model 4, we choose to use the LMCCC model as a basis for this analysis. We do note that the *theoretical results* presented here also hold for model 4 and, if the carrying capacity remains constant, for model 5 also.

Our analysis will concentrate on the fixed frequency screening policy. This policy assigns to each village a fixed time interval for consecutive screening rounds based on the village’s characteristics. As the policy is relatively easy to understand and implement, it has been the basis for guideline documents for HAT control. For example, the WHO recommends a screening interval of one year for villages reporting at least one case in the past three years, and an interval of 3 years for villages that did not report a case in the last three years, but did report at least one case during the past five years [1].

In the first part of this section, we mathematically analyze the impact of a fixed screening policy for a given village and investigate the screening frequency required to *eradicate* HAT in that village. As mentioned in the introduction, we define that HAT is eradicated in the long term if the expected prevalence level goes to zero in the long term.

A shorter term objective is to *eliminate* HAT, where elimination is defined as having at most one new case per 10000 persons per year [1, 7]. For example, the WHO’s roadmap towards elimination of HAT states the aim to eliminate (gambiense) HAT as a public health problem by 2020—which is defined as having less than one new case per 10000 inhabitants in at least 90% of the disease foci [1]—and to reach worldwide elimination by 2030. The second part of this section presents analytical results about the time needed to reach elimination and about the screening frequency requirements for reaching elimination *within a given time frame*. As our models consider expected prevalence instead of incidence, we redefine elimination as “reaching an expected prevalence level of one case per 10000”. We argue that the times and efforts required to reach this elimination target are practically suitable lower bounds on the times and efforts needed to reach the WHO’s targets. First, incidence and prevalence levels are argued to be “comparable” for HAT if mobile units visit afflicted areas infrequently [16]. If mobile teams visit the areas more frequently, incidence will only become larger compared to prevalence and the prevalence level target will be easier to achieve than the incidence level target (e.g., under the assumption that the fraction of flies infected is proportional to the fraction of humans infected, this follows directly from the epidemic model presented by Rogers [22]). Second, even if the *expected* prevalence level is below the defined threshold level, the intrinsic variability of the HAT epidemic may induce an *actual* prevalence level that exceeds this threshold.

Throughout this section, we consider an imaginary village with a constant carrying capacity *K*. (For sake of conciseness we omit the subscript *v* in this section). Furthermore, we assume a constant participation level *p*_*vn*_ = *p*, 0 < *p* < 1, and a fixed screening interval *τ*. The expected prevalence level at the beginning of the time horizon is denoted by *f*(0), *f*(0) > 0. Finally, recall that *s*, 0 < *s* < 1, denotes the sensitivity level.

#### Screening Requirements for Eradication

Let ${\bar{f}}_{n,n+1}$ denote the expected average prevalence level faced between screening rounds *n* and *n* + 1. In S2 Text, we prove the following results, which imply that the expected average HAT prevalence level will go to zero if the screening interval *τ* is at most $\frac{-\log(1-p\cdot s)}{\kappa }$ years, and that it will go to some strictly positive level otherwise:

**Proposition 1**. *If* $\tau \leq \frac{-\log(1-p\cdot s)}{\kappa }$, *then* $\lim_{n\to \infty }{\bar{f}}_{n,n+1}=0$.

**Proposition 2**. *If* $\tau >\frac{-\log(1-p\cdot s)}{\kappa }$, *then* $\lim_{n\to \infty }{\bar{f}}_{n,n+1}=K(\frac{\log(1-p\cdot s)}{\kappa \cdot \tau }+1)$.

**Fig 5:**
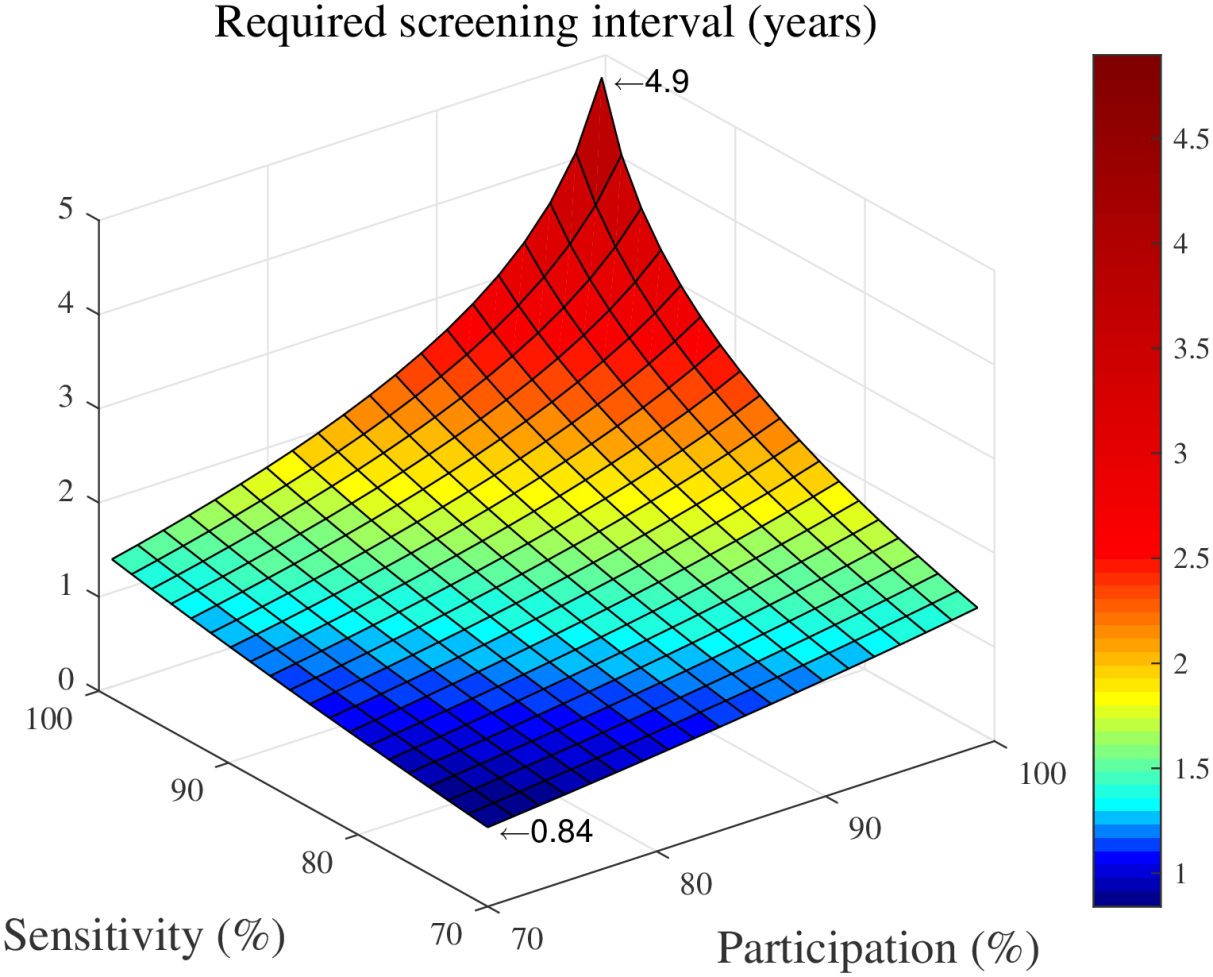
Screening interval required to reach elimination, for different levels of participation in the screening activities, and different levels of the sensitivity of the diagnostic test(s) performed.

A first insight provided by these propositions is that the eradication threshold for the screening interval strongly depends on *p* and *s*: the level of participation in the screening activities and the sensitivity of the diagnostic test. To illustrate this, Fig 5 shows the screening interval required for eradication for a range of values of the participation level and the sensitivity level. For example, if both values equal 70%, the required screening interval is 0.84 years (approximately once per 10 months). In case of participation and sensitivity levels of 99%, the required interval equals 4.9 years. This reveals the tremendous benefits of maximizing these values.

A second main insight is that annual screening, as currently recommended for endemic areas [1], only leads to eradication if the case detection fraction—defined as participation times sensitivity—exceeds 55%. For example, this is realized when the participation and sensitivity levels are both at least 75%. Furthermore, under realistic current sensitivity and participation levels—92.5% based on [4], and 70% based on our dataset, respectively—eradication requires a screening frequency of at least once per 1.30 years (approximately 15 months).

A final insight is that the eradication threshold does not depend on the village’s carrying capacity, meaning that the same upper bound on the screening frequency holds for all endemic areas. This can be understood by observing that changing the carrying capacity just changes the prevalence level with a constant factor at each point of time (see Eq (4)). Consequently, also the long term average prevalence changes with this constant factor, and hence remains zero if and only if the eradication threshold is met. We note that this does not imply that the disease burden, as measured by the number of person-years of illness, does not depend on the village’s carrying capacity. In contrast, our results in S2 Text. reveal that, for a given screening policy, the disease burden scales linearly with the carrying capacity.

#### Screening Requirements for Elimination

Let $\alpha =\frac{1}{1-p\cdot s}e^{-\kappa \cdot \tau }$, $\beta =\frac{p\cdot s}{1-p\cdot s}$, and $A_{0}=\frac{K}{f(0)}-1$. In S3 Text, we prove the following result on the time till the expected prevalence level hits a predefined level *C* in case of a screening interval of length *τ*:

**Proposition 3**. *If* *f*(0) > *C* *and* $\tau \leq \frac{-\log(1-p\cdot s)}{\kappa }$, the expected prevalence level is smaller than or equal to C for the first time after screening round n*, *where*

$$n^{*}=\left\{\begin{array}{cc} \left\lceil {}^{\alpha }\log\left(\frac{\frac{K}{C}-1+\frac{\beta }{\alpha -1}}{A_{0}+\frac{\beta }{\alpha -1}}\right)\right\rceil \; & if\;\alpha >1\\ \left\lceil \frac{\frac{K}{C}-1-A_{0}}{\beta }\right\rceil \; & if\;\alpha =1 \end{array}\right.$$ (14)

This result enables us to estimate the time needed to reach the expected prevalence level of 1/10000 for a given village. For example, let us consider a “typical village” with a current long term prevalence level ($\tilde{x}$) of 5/1000 and let us assume that $\tilde{\mu }=1$ (the median historic screening frequency in our dataset) and that $f\left(0\right)=0.5\tilde{x}$. Next, let us use the fitted LMCCC model described in Table 1 to estimate the village’s carrying capacity. Then, if annual screening is maintained and under the estimated current sensitivity and participation levels, the estimated time to elimination is 10 years.

**Fig 6:**
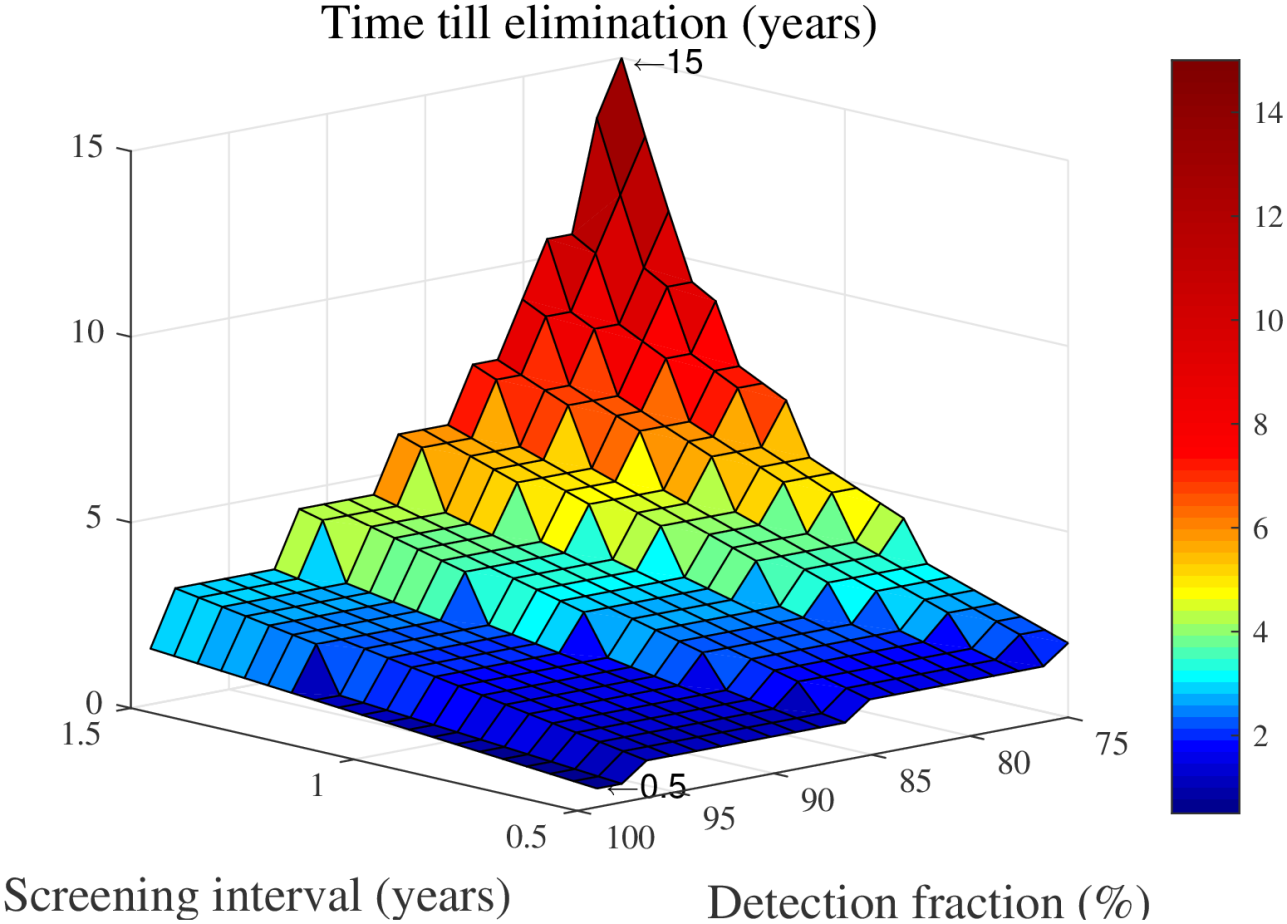
Time needed to reach elimination in a “typical village” (current prevalence level of 5/1000), for different levels of the case detection fraction (*p* ⋅ *s*) and different screening intervals.

Fig 6 provides some more insight into the relationship between the case detection fraction, the screening interval and the number of years needed to reach elimination. We see that the time to elimination ranges from 0.5 years (detection fraction: 99%, screening interval: 6 months) to 15 years (detection fraction: 75%, screening interval: 1.5 years) and that the detection fraction has a substantial impact on the time to elimination. For example, in case of a screening interval of 1.5 years, the time to elimination decreases from 15 years to 4.5 years when the case detection fraction increases from 75% to 90%. As explained, we consider the times shown here to be lower bounds on the times needed to reach the WHO’s targets, which is in line with earlier findings [10, 16].

Now, suppose that we would like to determine the screening interval needed to let the prevalence level cross the boundary *C* *within* *T* *years*. From the results stated in S2 Text. it follows that this is equivalent to finding n*: the smallest number of screening rounds such that $A_{n^{*}}\geq \frac{K}{C}-1$. Note that, under the fixed frequency policy, performing *n* screening rounds in *T* years implies a screening interval of $\frac{T}{n}$ years. In the following result, we assume that the first screening round is performed at time $\frac{T}{n}$ (and hence that the last is performed at time *T*):

**Lemma 1**. *Given that* $\tau =\frac{T}{n}\leq \frac{-\log(1-p\cdot s)}{\kappa }$, *then* lim_*n* → ∞_ *A*_*n*_ = ∞ *and the sequence* {*A*_*n*_} *is monotonically increasing in n*.

This lemma justifies the use of a simple search algorithm for finding the number of screening rounds to perform in the next *T* years, given the target prevalence level *C*. For example, this number can be found by increasing the value of *n* till *A*_*n*_ exceeds the threshold value $\frac{K}{C}-1$.

We use this approach to determine the screening interval required to reach elimination *within 5 years from now* for different levels of the case detection fraction and different current long term prevalence levels ($\tilde{x}$) of an imaginary village. Again, we use the fitted LMCCC model described in Table 1 for estimating the village’s carrying capacity and assume that $\tilde{\mu }=1$ and that $f\left(0\right)=0.5\tilde{x}$.

**Fig 7:**
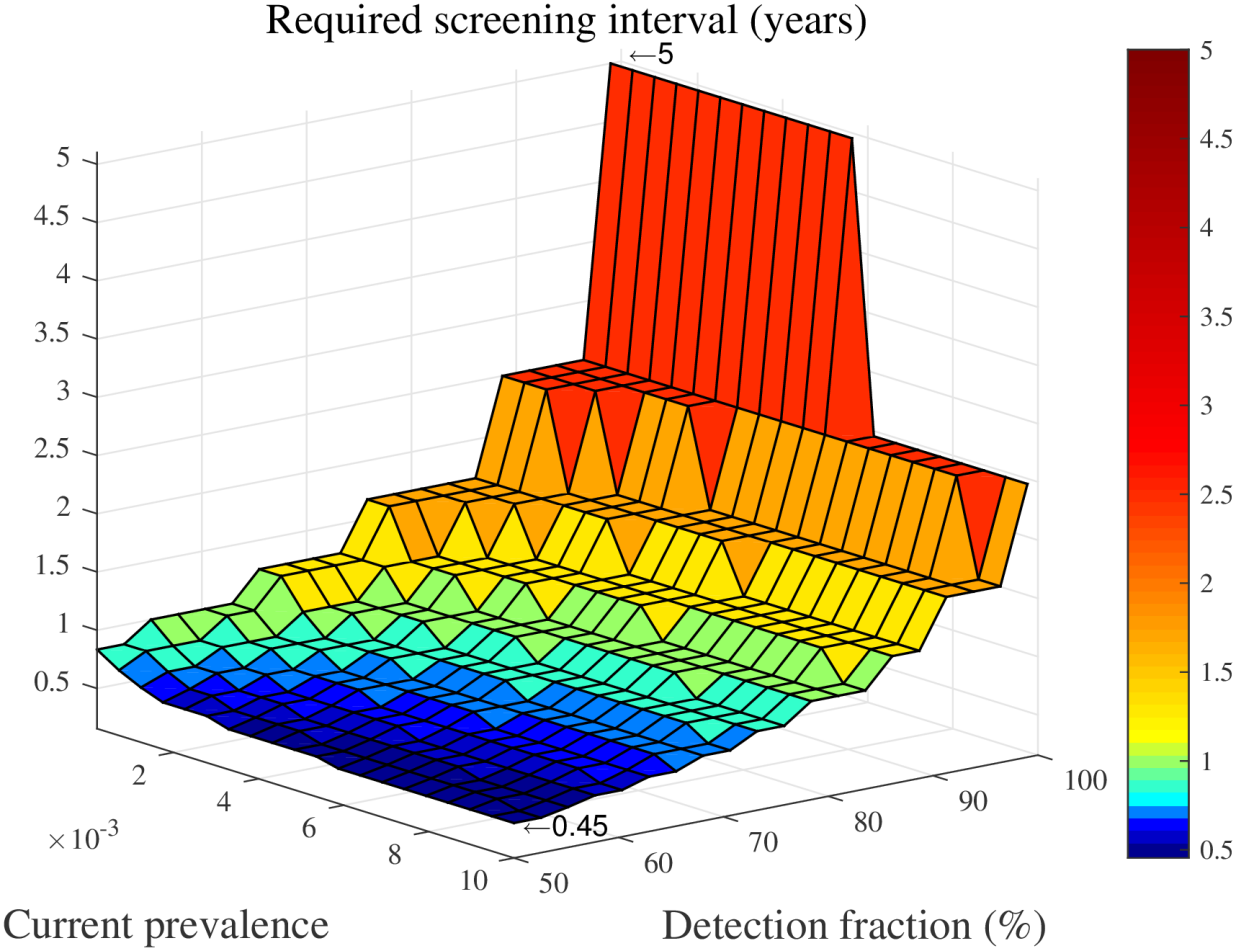
Screening interval required to reach elimination within five years, for different levels of the case detection fraction (*p* ⋅ *s*) and different current long term prevalence levels ($\tilde{x}$).

Fig 7 depicts the results. We see that the number of screening rounds needed to reach elimination within five years ranges from 1 (i.e. *τ* = 5) to 11 (i.e. *τ* = 0.45). Another observation is that *annual* screening only leads to elimination within five years if the case detection fraction is very high—roughly above 75%—or if the current prevalence level is very low—roughly below 1/1000. For the reason stated before, we consider this to be a lower bound on the efforts needed to reach the WHO’s targets within five years.

## Discussion

This paper introduces and analyzes five models for predicting HAT prevalence in a given village based on past observed prevalence levels and past screening activities in that village. Based on the quality of prevalence level predictions in 143 villages in Kwamouth (DRC), and based on the theoretical foundation underlying the models, we conclude that variants of the logistic model—a model inspired by the SIS model—are most practically suitable for predicting HAT prevalence levels. Sensitivity analyses show that this conclusion is very robust with respect to assumptions about participation levels, the sensitivity of the diagnostic test, or the initialization value of the prevalence curves are violated. Second, we demonstrate the applicability of one variant of the logistic model to analyze the effectiveness of the fixed frequency screening policy, which assigns to each village a fixed time interval for consecutive screening rounds.

Due to the intrinsic variability of the HAT epidemic, observed prevalence levels will generally deviate significantly from predicted prevalence levels. We strongly believe, however, that this does not render predictions worthless in the context of planning decisions. In contrast, a major contribution of our models is that they indicate the *expected* disease burden in different villages and can hence be applied to develop planning policies that aim to minimize the total *expected* disease burden for the villages considered.

Our analysis of the fixed frequency screening policy reveals that *eradication* of HAT is to be expected in the long term when the screening interval is smaller than a given threshold. This threshold strongly depends on the case detection fraction: the fraction of cases who participate in the screening rounds *and* are detected by the diagnostic tests. Under current conditions, we estimate the threshold to be approximately 15 months. This suggests that annual screening, as recommended by the WHO for endemic areas, will eventually lead to eradication. More specifically, our model predicts that annual screening will lead to eradication if the case detection fraction exceeds 55%.

The logistic model also reveals expressions for the time needed to reach the more short term target of *eliminating* HAT and for the screening interval required to eliminate HAT *within a given time frame*. These suggest that it takes 10 years to eliminate HAT in a village or focus with a prevalence of 5/1000 (under current conditions and annual screening). Furthermore, we estimate that it is only feasible to reach elimination within five years if the case detection fraction is very high—roughly above 75%—or if the current prevalence level is very low—roughly below 1/1000. We argue that these figures are practically suitable lower bounds on the time or efforts needed to reach the WHO’s targets for elimination.

Our results on requirements for eradication or elimination are based on a deterministic model, which begs to question their validity for reality, where events are stochastic. We note, however, that we model the *expected* behavior of a stochastic system, and hence that our results also hold *in expectation* for the stochastic system. On the other hand, we acknowledge that our models are not perfect. For example, we neglect interaction effects between neighboring villages. It would therefore be interesting and relevant to investigate whether our results can be reproduced by a validated simulation model.

A necessary condition for the applicability of our prediction models is that data about possible past HAT cases are available. Obviously, they thereby fail to identify endemic villages that have never been visited by a screening team. Different types of models, making use of different types of data—such as vegetation data (see e.g. [36]) or data about cases in nearby areas—are needed for these villages. A second limitation of the models is that they have been tested on a set of villages for which the screening interval was relatively short. Consequently, it is hard to establish the behavior of the epidemic if much larger screening intervals are used or if screening operations would be abandoned. Since datasets that do reveal this behavior are lacking, fitting the models to an appropriate dataset obtained by a validated simulation model would be a promising direction for future research.

We consider it likely that the speed of convergence to an epidemic equilibrium, as indicated by the parameter *κ*, differs per disease focus. This may be due to differences in epidemiological conditions, such as the presence of an animal reservoir and the specific tsetse subspecies living in a focus. These differences induce a need for developing and analyzing a cost-effective control strategy that takes differences between foci into account, as also recognized by Simarro et al. [37]. Repeating our analyses for different disease foci to investigate differences in requirements for elimination and eradication would therefore be highly relevant.

Our analyses revealed that the effectiveness of screening operations strongly depends on the participation level in the screening activities and the sensitivity of the diagnostic tests. Using screening algorithms with a higher sensitivity or case finding procedures that increase participation levels therefore seems to be very effective. For example, Robays et al. [23] list several combinations of diagnostic tests with different sensitivity levels, and a more acceptable case finding procedure is currently being piloted in the DRC. Using our model to investigate the cost-effectiveness of different control strategies, defining the screening frequencies as well as the screening procedures and diagnostic tests used, would be a promising direction for future research.

The results on the fixed frequency screening policy are all concerned with the screening frequency or the time needed to reach certain *targets*. While at a strategic level, these results might be interesting, at a tactical or operational level, screening frequency decisions are constrained by resource availability. Consequently, policies providing effective, acceptable, and practically suitable recommendations on how to allocate *available screening capacity* over the villages at risk are of much higher relevance at these levels. Literature about this allocation problem is however absent, as also observed by the WHO [1], so that future research addressing this topic is much needed.

## Supporting Information

S1 Text Notes data cleanup. (PDF)

S2 Text Proofs on screening requirements for eradication. (PDF)

S3 Text Proofs on screening requirements for elimination. (PDF)

S1 Table Table of notations. (PDF)

S2 Table Results for maximum likelihood approaches. (PDF)

S3 Table Sensitivity analysis on the sensitivity level and participation level. (PDF)

S4 Table Sensitivity analysis on the initial value assumption. (PDF)

S1 Fig Prediction errors. (PDF)

## References

[1] WHO. Control and surveillance of human African trypanosomiasis.; 2015. 984. 24552089

[2] Simarro PP, Cecchi G, Franco JR, Paone M, Ruiz-Postigo ADJA, Fèvre EM, et al Estimating and mapping the population at risk of sleeping sickness. PLoS Negl Trop Dis. 2012;6(10):e1859 10.1371/journal.pntd.0001859 23145192 PMC3493382

[3] WHO. Human African trypanosomiasis (sleeping sickness), factsheet 259; 2015. Available from: www.who.int/mediacentre/factsheets/fs259/en/.

[4] Brun R, Blum J, Chappuis F, Burri C. Human African trypanosomiasis. Lancet. 2010;375(9709):148–159. 10.1016/S0140-6736(09)60829-1 19833383

[5] Checchi F, Filipe JAN, Haydon DT, Chandramohan D, Chappuis F. Estimates of the duration of the early and late stage of gambiense sleeping sickness. BMC Infect Dis. 2008;8(1):16 10.1186/1471-2334-8-16 18261232 PMC2259357

[6] Fevre EM, Picozzi K, Jannin J, Welburn SC, Maudlin I. Human African trypanosomiasis: epidemiology and control. Adv Parasitol. 2006;61:167–221. 10.1016/S0065-308X(05)61005-6 16735165

[7] WHO. Report of a WHO meeting on elimination of African trypanosomiasis (Trypanosoma brucei gambiense); 2013.

[8] Hasker E, Lutumba P, Mumba D, Lejon V, Büscher P, Kande V, et al Diagnostic accuracy and feasibility of serological tests on filter paper samples for outbreak detection of Tb gambiense human African trypanosomiasis. Am J Trop Med Hyg. 2010;83(2):374 10.4269/ajtmh.2010.09-0735 20682885 PMC2911188

[9] Mpanya A, Hendrickx D, Vuna M, Kanyinda A, Lumbala C, Tshilombo V, et al Should I get screened for sleeping sickness? A qualitative study in Kasai province, Democratic Republic of Congo. PLoS Negl Trop Dis. 2012;6(1):e1467 10.1371/journal.pntd.0001467 22272367 PMC3260312

[10] Rock KS, Torr SJ, Lumbala C, Keeling MJ. Quantitative evaluation of the strategy to eliminate human African trypanosomiasis in the Democratic Republic of Congo. Parasit Vectors. 2015;8(1):1–13. 10.1186/s13071-015-1131-8 26490248 PMC4618948

[11] Moore A, Richer M. Re-emergence of epidemic sleeping sickness in southern Sudan. Trop Med Int Health. 2001;6(5):342–347. 10.1046/j.1365-3156.2001.00714.x 11348529

[12] Paquet C, Castilla J, Mbulamberi D, Beaulieu MF, Moren A. Trypanosomiasis from Trypanosoma brucei gambiense in the center of north-west Uganda. Evaluation of 5 years of control (1987–1991). Bull Soc Pathol Exot. 1994;88(1):38–41. 7787452

[13] Van Nieuwenhove S, Betu-Ku-Mesu VK, Diabakana PM, Declercq J, Bilenge CMM. Sleeping sickness resurgence in the DRC: the past decade. Trop Med Int Health. 2001;6(5):335–341. 10.1046/j.1365-3156.2001.00731.x 11348528

[14] Ruiz JA, Simarro PP, Josenando T. Control of human African trypanosomiasis in the Quicama focus, Angola. Bulletin of the World Health Organization. 2002;80(9):738–745. 12378293 PMC2567610

[15] Simarro P, Sima F, Mir M, Mateo M, Roche J. Control of human African trypanosomiasis in Luba in equatorial Guinea: evaluation of three methods. Bulletin of the World Health Organization. 1990;69(4):451–457. PMC2393243 1934239

[16] Stone CM, Chitnis N. Implications of heterogeneous biting exposure and animal hosts on Trypanosomiasis brucei gambiense transmission and control. PLoS Comput Biol. 2015;11(10):e1004514 10.1371/journal.pcbi.1004514 26426854 PMC4591123

[17] Funk S, Nishiura H, Heesterbeek H, Edmunds WJ, Checchi F. Identifying transmission cycles at the human-animal interface: the role of animal reservoirs in maintaining gambiense human African trypanosomiasis. PLoS Comput Biol. 2013;9(1):e1002855 10.1371/journal.pcbi.1002855 23341760 PMC3547827

[18] Medlock J, Atkins KE, Thomas DN, Aksoy S, Galvani AP. Evaluating paratransgenesis as a potential control strategy for African trypanosomiasis. PLoS Negl Trop Dis. 2013;7(8):e2374 10.1371/journal.pntd.0002374 23967363 PMC3744416

[19] Artzrouni M, Gouteux JP. A model of Gambian sleeping sickness with open vector populations. Math Med Biol. 2001;18(2):99–117. 10.1093/imammb/18.2.99 11453470

[20] Chalvet-Monfray K, Artzrouni M, Gouteux JP, Auger P, Sabatier P. A two-patch model of Gambian sleeping sickness: application to vector control strategies in a village and plantations. Acta Biotheor. 1998;46(3):207–222. 10.1023/A:1001776926167 10220868

[21] Jusot JF, De Vlas SJ, Van Oortmarssen GJ, De Muynck A. Apport d’un modele mathématique dans le controle d’une parasitose: cas de la trypanosomiase humaine africaine a Trypanosoma brucei gambiense. Ann Soc Belg Med Trop. 1995;75:257–272. 8669973

[22] Rogers DJ. A general model for the African trypanosomiases. Parasitology. 1988;97(01):193–212. 10.1017/S0031182000066853 3174235

[23] Robays J, Bilengue MMC, Stuyft PVd, Boelaert M. The effectiveness of active population screening and treatment for sleeping sickness control in the Democratic Republic of Congo. Trop Med Int Health. 2004;9(5):542–550. 10.1111/j.1365-3156.2004.01240.x 15117297

[24] Artzrouni M, Gouteux JP. Control strategies for sleeping sickness in Central Africa: a model-based approach. Trop Med Int Health. 1996;1(6):753–764. 10.1111/j.1365-3156.1996.tb00107.x 8980586

[25] Abeku TA, De Vlas SJ, Borsboom G, Teklehaimanot A, Kebede A, Olana D, et al Forecasting malaria incidence from historical morbidity patterns in epidemic-prone areas of Ethiopia: a simple seasonal adjustment method performs best. Trop Med Int Health. 2002;7(10):851–857. 10.1046/j.1365-3156.2002.00924.x 12358620

[26] Allard R. Use of time-series analysis in infectious disease surveillance. Bulletin of the World Health Organization. 1998;76(4):327 9803583 PMC2305771

[27] Franses PH. Time series models for business and economic forecasting. Cambridge university press; 1998.

[28] Diekmann O, Heesterbeek J, Metz JA. On the definition and the computation of the basic reproduction ratio R 0 in models for infectious diseases in heterogeneous populations. J Math Biol. 1990;28(4):365–382. 10.1007/BF00178324 2117040

[29] Davis S, Aksoy S, Galvani A. A global sensitivity analysis for African sleeping sickness. Parasitology. 2011;138(04):516–526. 10.1017/S0031182010001496 21078220 PMC3282146

[30] Simarro PP, Cecchi G, Paone M, Franco JR, Diarra A, Ruiz JA, et al The Atlas of human African trypanosomiasis: a contribution to global mapping of neglected tropical diseases. Int J Health Geogr. 2010;9(1):57–75. 10.1186/1476-072X-9-57 21040555 PMC2988709

[31] Welburn SC, Molyneux DH, Maudlin I. Beyond tsetse–implications for research and control of human African trypanosomiasis epidemics. Trends Parasitol. 2016;32(3):230–241. 10.1016/j.pt.2015.11.008 26826783

[32] Heij C, De Boer P, Franses PH, Kloek T, Van Dijk HK, et al Econometric methods with applications in business and economics. Oxford University Press; 2004.

[33] Checchi F, Cox AP, Chappuis F, Priotto G, Chandramohan D, Haydon DT, et al Prevalence and under-detection of gambiense human African trypanosomiasis during mass screening sessions in Uganda and Sudan. Parasit Vectors. 2012;5:157 10.1186/1756-3305-5-157 22871103 PMC3430581

[34] Pépin J, Méda H. The epidemiology and control of human African trypanosomiasis. Adv Parasitol. 2001;49:71–132. 10.1016/S0065-308X(01)49038-5 11461032

[35] Truc P, Lejon V, Magnus E, Jamonneau V, Nangouma A, Verloo D, et al Evaluation of the micro-CATT, CATT/Trypanosoma brucei gambiense, and LATEX/T. b. gambiense methods for serodiagnosis and surveillance of human African trypanosomiasis in West and Central Africa. Bull World Health Organ. 2002;80(11):882–886. 12481210 PMC2567684

[36] Rogers DJ. Satellites, space, time and the African trypanosomiases. Adv Parasitol. 2000;47:129–171. 10.1016/S0065-308X(00)47008-9 10997206

[37] Simarro P, Franco J, Diarra A, Ruiz Postigo J, Jannin J. Diversity of human African trypanosomiasis epidemiological settings requires fine-tuning control strategies to facilitate disease elimination. Res Rep Trop Med. 2013;4:1–6. 30100778 10.2147/RRTM.S40157 PMC6067614

