# Supplementary material for: Forecasting Human African Trypanosomiasis Prevalences from Population Screening Data Using Continuous Time Models
Source: PLoS Comput Biol. 2016 Sep 22;12(9):e1005103. doi: 10.1371/journal.pcbi.1005103 (PMC5033383; doi:10.1371/journal.pcbi.1005103)
Supplement: S1 Text — (PDF) [file pcbi.1005103.s001.pdf]

S1 Text

Notes Data Cleanup. The following rules were used for cleaning up the database:

- If only the year of screening was reported, assume that it took place at July 1st.
- If the month of screening was not reported, assume that it took place in July.
- If the day of screening was not reported or invalid (e.g., 0), assume that it took place at the 1st day of the month.
- Only select data between 01-01-2004 and 31-12-2013 (there are some data about 2014, but these are incomplete).
- If the dates of two screening rounds are at most three months apart, we regard them as one screening round.
- If two screening rounds are entered in the same cell ($>$ 3 months apart), but the number of people screened is entered only once, assume that the number of people screened is the same for both rounds.
- If a screening round took multiple days, choose the first day as the screening date.
- If the number of people screened in a screening round is not reported, assume it to be equal to the average number of people screened in the rounds for which this number *is* reported.
