# Supplementary material for: Forecasting Human African Trypanosomiasis Prevalences from Population Screening Data Using Continuous Time Models
Source: PLoS Comput Biol. 2016 Sep 22;12(9):e1005103. doi: 10.1371/journal.pcbi.1005103 (PMC5033383; doi:10.1371/journal.pcbi.1005103)
Supplement: S2 Text — (PDF) [file pcbi.1005103.s002.pdf]

S2 Text

Proofs on Screening Requirements for Eradication. For sake of conciseness we omit the subscript v in this section. Before we state our proofs, let us make some observations about the expected prevalence level immediately after the nth screening round:

$$f(S_n^+) = \frac{K}{1 + A_n} \tag{1}$$

Let us specifically focus on the parameter A_n. Note that a larger value of A_n implies a lower prevalence level after the screening round and vice versa. Recall that the impact of a screening round is estimated as (see main text):

$$f_v(S_{vn}^+) = (1 - p_{vn} \cdot s)\, f_v(S_{vn}^-),$$

This implies for any $n \geq 1$:

$$\begin{aligned}
A_n &= \frac{K}{f(S_n^+)} - 1 &(2)\\
&= \frac{K}{(1 - p \cdot s) \cdot f(S_n^-)} - 1 &(3)\\
&= \frac{K}{(1 - p \cdot s) \cdot \frac{K}{1 + A_{n-1} \cdot e^{-\kappa \cdot \tau}}} - 1 &(4)\\
&= \alpha \cdot A_{n-1} + \beta &(5)\\
&= \alpha^n \cdot A_0 + \sum_{i=1}^{n} \alpha^{n-i} \cdot \beta &(6)\\
&= \begin{cases} \alpha^n \cdot A_0 + \beta \frac{\alpha^n - 1}{\alpha - 1} & \text{if } \alpha \neq 1 \\ A_0 + n \cdot \beta & \text{if } \alpha = 1 \end{cases} &(7)
\end{aligned}$$

Here, $\alpha = \frac{1}{1-p \cdot s} e^{-\kappa \cdot \tau}$, $\beta = \frac{p \cdot s}{1 - p \cdot s}$, and $A_0 = \frac{K}{f(0)} - 1$. Note that α, β, and A_0 are strictly positive. These observations and definitions allow us to prove the following results.

Lemma 1. *If $\tau \leq \frac{-\log(1-p\cdot s)}{\kappa}$, then $\lim_{n\to\infty} A_n = \infty$ and A_n is discretely convex and increasing in n.*

Proof. First, rewriting yields that the condition $\tau \leq \frac{-\log(1-p\cdot s)}{\kappa}$ is equivalent to the condition $\alpha = \frac{1}{1-p\cdot s} e^{-\kappa \cdot \tau} \geq 1$. The result now follows immediately from Eq. (7) and by the observations that $A_0 > 0$ and $\beta > 0$. If $\alpha > 1$ these imply that:

$$\lim_{n\to\infty} A_n > \lim_{n\to\infty} \alpha^n A_0 = \infty \tag{8}$$

Furthermore, if $\alpha = 1$, it holds that:

$$\lim_{n\to\infty} A_n = \lim_{n\to\infty} A_0 + n \cdot \beta = \infty \tag{9}$$

The discrete convexity of A_n is trivial for the case that $\alpha = 1$, since A_n is linear in n in this case. If $\alpha > 1$, the convexity follows from the fact that every function having the form $c \cdot \alpha^n + d$ is convex in n for $c \geq 0$ and $\alpha \geq 0$. Finally, the fact that A_n is increasing in n follows directly from Eq (6).

□

Lemma 2. *If* $\tau > \frac{-\log(1-p\cdot s)}{\kappa}$*, then then the sequence* $\{A_n\}$ *converges monotonically to* $A = \frac{\beta}{1-\alpha}$*.*

Proof. First, rewriting yields that the condition $\tau > \frac{-\log(1-p\cdot s)}{\kappa}$ is equivalent to the condition $\alpha = \frac{1}{1-p\cdot s}e^{-\kappa\cdot\tau} < 1$. The fact that the sequence converges to A now follows immediately by taking the limit in Eq. (7):

$$\lim_{n\to\infty} A_n = \lim_{n\to\infty} \alpha^n \cdot A_0 + \beta\frac{\alpha^n - 1}{\alpha - 1} \tag{10}$$
$$= \frac{\beta}{1-\alpha} = A \tag{11}$$

To prove that A_n converges *monotonically* to A, we distinguishing two cases: $A_{n-1} < A$ and $A_{n-1} > A$, (it is obvious for $A_{n-1} = A$). First, consider the case that $A_{n-1} < A$. We show that $A_n < A$, and that $A_n - A_{n-1} > 0$, implying monotonicity. The first claim is implied by Eq. (6):

$$A_n = \alpha \cdot A_{n-1} + \beta \tag{12}$$
$$< \alpha \cdot \frac{\beta}{1-\alpha} + \beta \tag{13}$$
$$= \frac{\beta}{1-\alpha} = A \tag{14}$$

To prove the second claim we make use of the given that $\alpha < 1$:

$$A_n - A_{n-1} = \alpha \cdot A_{n-1} + \beta - A_{n-1} \tag{15}$$
$$= (\alpha - 1)\, A_{n-1} + \beta \tag{16}$$
$$> (\alpha - 1)\, \frac{\beta}{1-\alpha} + \beta = 0 \tag{17}$$

Next, consider the case that $A_{n-1} > A$. Using on exactly the same reasoning as the one presented above, it follows that $A_n > A$ as well, and that $A_n - A_{n-1} < 0$.

This completes the proof. □

Now we have established the (long term) behavior of the parameter A_n, let us consider the implications for the (long term) prevalence level. Recall that $\bar{f}_{n,n+1}$ denotes the expected average prevalence level faced between screening rounds n and $n+1$, which is calculated as:

$$\bar{f}_{n,n+1} = \frac{1}{\tau}\int_0^{\tau} \frac{K}{1 + A_n \cdot e^{-\kappa\cdot t}} dt \tag{18}$$
$$= \frac{K}{\kappa\cdot\tau}\log\left(\frac{A_n + e^{\kappa\cdot\tau}}{A_n + 1}\right) \tag{19}$$

Using this definition, we now prove the desired results:

Proposition 1. *If* $\tau \le \frac{-\log(1-p\cdot s)}{\kappa}$*, then* $\lim_{n\to\infty} \bar{f}_{n,n+1} = 0$*.*

Proof. From Lemma 1 we know that $\lim_{n\to\infty} A_n = \infty$. Hence, we derive that:

$$\lim_{n\to\infty} \bar{f}_{n,n+1} = \lim_{n\to\infty} \frac{K}{\kappa \cdot \tau} \log\left(\frac{A_n + e^{\kappa\cdot\tau}}{A_n + 1}\right) \tag{20}$$

$$= \frac{K}{\kappa \cdot \tau} \log(1) = 0 \tag{21}$$

□

Proposition 2. *If* $\tau > \frac{-\log(1-p\cdot s)}{\kappa}$, *then* $\lim_{n\to\infty} \bar{f}_{n,n+1} = K\left(\frac{\log(1-p\cdot s)}{\kappa\cdot\tau} + 1\right)$.

Proof. From Lemma 2 we know that $\lim_{n\to\infty} A_n = A = \frac{\beta}{1-\alpha}$. Hence, we derive that:

$$\lim_{n\to\infty} \bar{f}_{n,n+1} = \lim_{n\to\infty} \frac{K}{\kappa \cdot \tau} \log\left(\frac{A_n + e^{\kappa\cdot\tau}}{A_n + 1}\right) \tag{22}$$

$$= \frac{K}{\kappa \cdot \tau} \log\left((1 - p \cdot s)\, e^{\kappa\cdot\tau}\right) \tag{23}$$

$$= K\left(\frac{\log(1 - p \cdot s)}{\kappa \cdot \tau} + 1\right) \tag{24}$$

□
