# Supplementary material for: Forecasting Human African Trypanosomiasis Prevalences from Population Screening Data Using Continuous Time Models
Source: PLoS Comput Biol. 2016 Sep 22;12(9):e1005103. doi: 10.1371/journal.pcbi.1005103 (PMC5033383; doi:10.1371/journal.pcbi.1005103)
Supplement: S3 Text — (PDF) [file pcbi.1005103.s003.pdf]

S3 Text

Proofs on Screening Requirements for Elimination. For sake of conciseness, we omit the subscript v in this section. Recall that $\alpha = \frac{1}{1-p\cdot s}e^{-\kappa\cdot\tau}$, $\beta = \frac{p\cdot s}{1-p\cdot s}$, that $A_0 = \frac{K}{f(0)} - 1$, and that α, β, and A_0 are strictly positive.

Proposition 3. *If $f(0) > C$ and $\tau \leq \frac{-\log(1-p\cdot s)}{\kappa}$, the expected prevalence level is smaller than or equal to C after screening round n^*, where*

$$n^* = \begin{cases} \left\lceil {}^{\alpha}\log\left(\frac{\frac{K}{C}-1+\frac{\beta}{\alpha-1}}{A_0+\frac{\beta}{\alpha-1}}\right)\right\rceil & \text{if } \alpha > 1 \\ \left\lceil \frac{\frac{K}{C}-1-A_0}{\beta} \right\rceil & \text{if } \alpha = 1 \end{cases} \tag{1}$$

Proof. First, rewriting yields that the condition $\tau \leq \frac{-\log(1-p\cdot s)}{\kappa}$ is equivalent to the condition $\alpha = \frac{1}{1-p\cdot s}e^{-\kappa\cdot\tau} \geq 1$. According to the LMCCC model, the first time the expected prevalence level crosses the boundary value C occurs immediately after a screening round. This is because the expected prevalence level only increases in the period between two screening rounds. Hence, we prove our theorem by determining the screening round $n^* : f(S^+_{n^*}) \leq C$. By the definition of $A_n = \frac{K}{f(S^+_n)} - 1$, we know that this is equivalent to determining $n^* : A_{n^*} \geq \frac{K}{C} - 1$. Now, substituting the following recurrence relation (see S2 Text)

$$A_n = \begin{cases} \alpha^n \cdot A_0 + \beta\frac{\alpha^n-1}{\alpha-1} & \text{if } \alpha > 1 \\ A_0 + n\cdot\beta & \text{if } \alpha = 1 \end{cases} \tag{2}$$

yields:

$$\alpha^n \cdot A_0 + \beta\frac{\alpha^n - 1}{\alpha - 1} \geq \frac{K}{C} - 1 \qquad \text{if } \alpha > 1 \tag{3}$$

$$A_0 + n\cdot\beta \geq \frac{K}{C} - 1 \qquad \text{if } \alpha = 1 \tag{4}$$

Rewriting these inequalities gives the desired result.

□

Lemma 1. *Given that $\tau = \frac{T}{n} \leq \frac{-\log(1-p\cdot s)}{\kappa}$, then $\lim_{n\to\infty} A_n = \infty$ and the sequence $\{A_n\}$ is monotonically increasing in n.*

Proof. To reflect that the value of α now depends on n, the number of screening rounds performed in the next T years, let us define α_n as:

$$\alpha_n = \frac{1}{1-p\cdot s}\cdot e^{-\kappa\frac{T}{n}} \tag{5}$$

Hence, rewriting the definition of A_n in terms of α_n yields:

$$A_n = \alpha_n^n \cdot A_0 + \sum_{i=1}^{n} \alpha_n^{n-i}\cdot\beta \tag{6}$$

In what follows, we make use of the fact that $\alpha_n \geq 1$, that $\alpha_n > \alpha_{n-1} > 0$ and hence that $\alpha_n^i > \alpha_{n-1}^i$. This property follows directly from the definition of α_n and from the

fact that $\kappa \cdot T$ is strictly positive. Using this result, the first part of the Lemma is shown by taking the limit for n:

$$\lim_{n\to\infty} A_n = \lim_{n\to\infty} \alpha_n^n \cdot A_0 + \sum_{i=1}^{n} \alpha_n^{n-i} \cdot \beta = \infty \tag{7}$$

The following inequality proves that $A_n - A_{n-1} > 0$, and hence that A_n increases monotonically with n:

$$A_n - A_{n-1} = A_0 \left(\alpha_n^n - \alpha_{n-1}^{n-1}\right) + \beta \left(\alpha_n^n + \sum_{i=1}^{n-2} \left(\alpha_n^i - \alpha_{n-1}^i\right)\right) > 0 \tag{8}$$

□
