# Supplementary material for: Forecasting Human African Trypanosomiasis Prevalences from Population Screening Data Using Continuous Time Models
Source: PLoS Comput Biol. 2016 Sep 22;12(9):e1005103. doi: 10.1371/journal.pcbi.1005103 (PMC5033383; doi:10.1371/journal.pcbi.1005103)
Supplement: S1 Table — (PDF) [file pcbi.1005103.s004.pdf]

S1 Table

Table of Notations.

Table 1. Table of Notations.

Village characteristics	
V	set of villages v
N_v	population size of village v
p_{vn}	participation fraction in the nth screening round for village v
S_{vn}	time at which the nth screening round in village v is performed
S_{vn}^{+}	moment immediately after the nth screening round in village v
S_{vn}^{-}	moment just before the nth screening round in village v
s_{vn}	time between the $n-1^{th}$ and the nth screening round in village v
$\delta_v^{-}(t)$	time since the last screening round in village v before time t
$\tilde{\mu}_v$	average screening frequency in village v during 5 consecutive years
$\bar{\mu}_v(S_{vn})$	average screening frequency in the 3 years prior to the nth screening round in village v
Prevalence level notations	
$x_v(t)$	prevalence level (fraction) in village v observed at time t
$f_v(t)$	expected prevalence level (fraction) in village v at time t
ε_v	random disturbance for the prevalence level (fraction) in village v
$\tilde{x}_v$	average observed prevalence level (fraction) in village v during 5 consecutive years
$\bar{x}_v(S_{vn})$	average observed prevalence level (fraction) in the 3 years prior to the nth screening round in village v
Prediction model notations	
α_v	fixed effect for village v
K_v	carrying capacity of village v
κ	growth rate parameter
A_{vn}	initial value parameter for screening round n in village v
e_{vn}	prediction error/ fitting deviation for screening round n in village v
w_{vn}	weight of observation n for village v
Disease characteristics	
r	yearly removal rate for HAT
R_0	basic reproduction number for HAT
s	sensitivity of the diagnostic test
Screening policy analysis notations	
τ	fixed time interval for screening rounds
$\bar{f}_{n,n+1}$	expected average prevalence level between the nth and the $(n+1)^{th}$ screening round
