# Supplementary material for: Forecasting Human African Trypanosomiasis Prevalences from Population Screening Data Using Continuous Time Models
Source: PLoS Comput Biol. 2016 Sep 22;12(9):e1005103. doi: 10.1371/journal.pcbi.1005103 (PMC5033383; doi:10.1371/journal.pcbi.1005103)
Supplement: S2 Table — (PDF) [file pcbi.1005103.s005.pdf]

S2 Table

Results for Maximum Likelihood Approaches.

Table 1. Predictive performance of the five models in terms of mean errors (ME), mean absolute errors (MAE), and mean relative errors (MRE) when they are fitted using a maximum likelihood approach assuming a Poisson, Beta-Binomial, or Negative Binomial distribution.

	Poisson			Beta-Binomial			Negative Binomial		
	ME	MAE	MRE	ME	MAE	MRE	ME	MAE	MRE
Model 1: LM	*	*	*	*	*	*	*	*	*
Model 2: FEM	-0.00080	0.00598	1.88	0.00168	0.00702	2.21	-0.000232	0.00523	1.65
Model 3: LMCCC	0.00132	0.00667	2.10	0.01056	0.01537	4.84	0.00689	0.01187	3.74
Model 4: rLMCCC	0.00575	0.01080	3.39	0.03352	0.03802	11.97	0.02248	0.02708	8.52
Model 5: LMVCC	-0.00007	0.00434	1.36	0.00639	0.00943	2.97	0.00194	0.00720	2.27

* Model could not be fitted: several observations require the calculation of $P(x_v(t) = y), y > 0$, whereas $f_v(t) = 0$. Since this probability equals 0 irrespective of the parameter estimates, the (log)likelihood could not be maximized.
