# Supplementary material for: Forecasting Human African Trypanosomiasis Prevalences from Population Screening Data Using Continuous Time Models
Source: PLoS Comput Biol. 2016 Sep 22;12(9):e1005103. doi: 10.1371/journal.pcbi.1005103 (PMC5033383; doi:10.1371/journal.pcbi.1005103)
Supplement: S3 Table — (PDF) [file pcbi.1005103.s006.pdf]

S3 Table

Sensitivity Analysis on the Sensitivity Level and Participation Level.

Table 1. Predictive performance of the logistic models in terms of mean errors (ME), mean absolute errors (MAE), and mean relative errors (MRE) when the real case detection fraction ($s \cdot p_{vn}$) deviates a fraction ρ from the values used in the baseline analysis. E.g., $\rho = -0.1$ means that the real fraction is 10% smaller than the presently used fraction. The best indicator values are in bold.

		$\rho = -0.2$			$\rho = -0.1$			$\rho = 0.05$	
	ME	MAE	MRE	ME	MAE	MRE	ME	MAE	MRE
Model 3: LMCCC	**−0.00018**	0.00530	1.67	**−0.00033**	0.00521	1.64	−0.00112	0.00463	1.46
Model 4: rLMCCC	0.00221	0.00728	2.29	0.00150	0.00674	2.12	**0.00014**	0.00577	1.82
Model 5: LMVCC	−0.00113	**0.00395**	**1.24**	−0.00136	**0.00382**	**1.20**	−0.00191	**0.00357**	**1.13**
