# Supplementary material for: Forecasting Human African Trypanosomiasis Prevalences from Population Screening Data Using Continuous Time Models
Source: PLoS Comput Biol. 2016 Sep 22;12(9):e1005103. doi: 10.1371/journal.pcbi.1005103 (PMC5033383; doi:10.1371/journal.pcbi.1005103)
Supplement: S4 Table — (PDF) [file pcbi.1005103.s007.pdf]

S4 Table

Sensitivity Analysis on the Initial Value Assumption.

Table 1. Predictive performance of the logistic models in terms of mean errors (ME), mean absolute errors (MAE), and mean relative errors (MRE) for different assumptions about ι, the parameter determining the expected prevalence level at the beginning of the time horizon: $f_v(0) = \iota\tilde{x}_v$.

	$\iota = 0.4$			$\iota = 0.6$			$\iota = 0.8$		
	ME	MAE	MRE	ME	MAE	MRE	ME	MAE	MRE
Model 3: LMCCC	−0.00086	0.00481	1.51	−0.00095	0.00475	1.50	−0.00096	0.00475	1.49
Model 4: rLMCCC	**0.00069**	0.00619	1.95	**0.00028**	0.00583	1.84	**0.00009**	0.00567	1.79
Model 5: LMVCC	−0.00171	**0.00395**	**1.16**	−0.00186	**0.00369**	**1.12**	−0.00194	**0.00350**	**1.10**
