# Supplementary material for: Forecasting Human African Trypanosomiasis Prevalences from Population Screening Data Using Continuous Time Models
Source: PLoS Comput Biol. 2016 Sep 22;12(9):e1005103. doi: 10.1371/journal.pcbi.1005103 (PMC5033383; doi:10.1371/journal.pcbi.1005103)
Supplement: S1 Fig — (PDF) [file pcbi.1005103.s008.pdf]

S1 Figure

Prediction Errors.

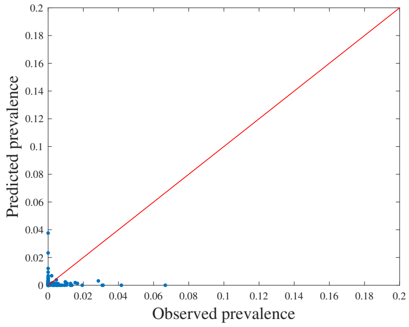

(A) LM model

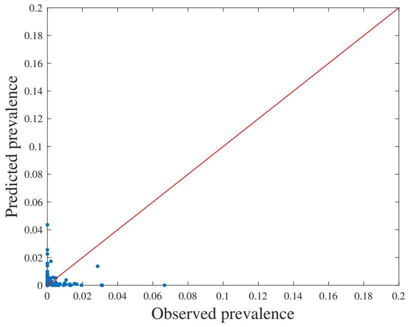

(B) FE model

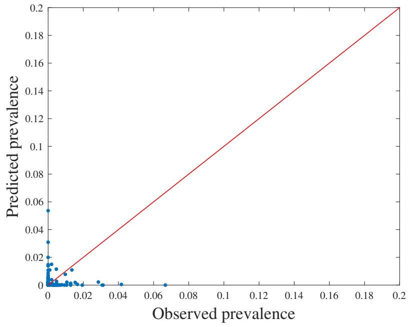

(C) LMCCC model

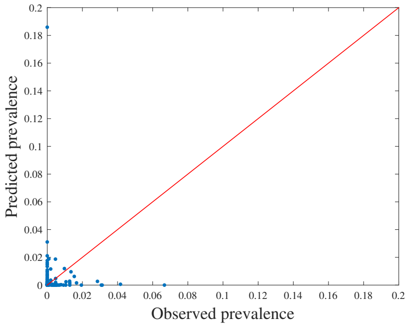

(D) rLMCCC model

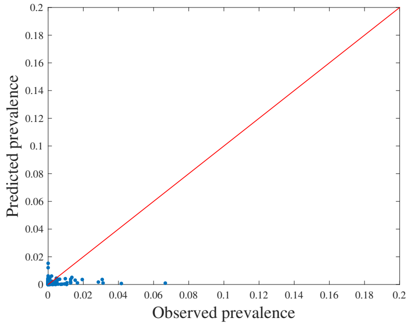

(E) LMVCC model

Predicted prevalence vs. observed prevalence for the 143 observations in the prediction sample.
